# Fiber‐Type Solar Cells, Nanogenerators, Batteries, and Supercapacitors for Wearable Applications

**DOI:** 10.1002/advs.201800340

**Published:** 2018-06-17

**Authors:** Sreekanth J. Varma, Kowsik Sambath Kumar, Sudipta Seal, Swaminathan Rajaraman, Jayan Thomas

**Affiliations:** ^1^ NanoScience Technology Center University of Central Florida Orlando FL 32826 USA; ^2^ Department of Materials Science & Engineering University of Central Florida Orlando FL 32826 USA; ^3^ Advanced Materials Processing Analysis Center University of Central Florida Orlando FL 32826 USA; ^4^ BRIDG—Bridging the Innovation Development Gap 200 NeoCity Way NeoCity FL 34744 USA; ^5^ Department of Electrical & Computer Engineering University of Central Florida Orlando FL 32826 USA; ^6^ CREOL The College of Optics and Photonics University of Central Florida Orlando FL 32816 USA

**Keywords:** integrated energy devices, Li‐ion batteries, solar cells, supercapacitors, wearable devices

## Abstract

Wearable electronic devices represent a paradigm change in consumer electronics, on‐body sensing, artificial skins, and wearable communication and entertainment. Because all these electronic devices require energy to operate, wearable energy systems are an integral part of wearable devices. Essentially, the electrodes and other components present in these energy devices should be mechanically strong, flexible, lightweight, and comfortable to the user. Presented here is a critical review of those materials and devices developed for energy conversion and storage applications with an objective to be used in wearable devices. The focus is mainly on the advances made in the field of solar cells, triboelectric generators, Li‐ion batteries, and supercapacitors for wearable device development. As these devices need to be attached/integrated with the fabric, the discussion is limited to devices made in the form of ribbons, filaments, and fibers. Some of the important challenges and future directions to be pursued are also highlighted.

## 1 Introduction

The concept of a self‐powered, self‐drying jacket and electromechanical shoes that lace themselves garnered significant public attention, since it was first depicted in the movie Back to the Future II, in 1989. However, it remained a cinematic fantasy until recently when considerable effort has been focused on developing electronic textiles and wearables. An exodus of electronic devices has occurred during the past couple of decades. These electronic devices have considerably simplified or eased the quality of human life and have had a significant impact on our day‐to‐day life, especially healthcare, communication, and transportation. Presently, smartphones perform numerous functions which were not possible a decade ago using just a mobile phone. We foresee that many of the functions currently performed by the smartphones will be bifurcated and executed by wearable devices worn or implanted into the body in the near future. In addition to the existing functions, these new wearable devices will perform many tasks that have not been imagined today. Conventional energy storage devices used in electronic devices are rigid and bulky and are a major limitation in accomplishing this mission. This places a fundamental need to develop new strategies for energy harvesting and storing that fulfill the requirements for current and future wearable devices.

In December of 2015, the world leaders from around 200 countries met in Paris to discuss climate change and global warming,^1^, ^2^ and find strategies to minimize the greenhouse effect mainly caused by the excessive use of fossil fuels as an energy source.^3^, ^4^, ^5^ However, energy is a basic necessity for the existence and growth of human civilization.^6^ Therefore, to reduce fossil fuel consumption, it is very necessary to utilize renewable energy sources and also make energy harvest easy and portable. The human consumption of energy in 2014 was about 159 000 TWh of which about 23 800 TWh was in the form of electricity. About 66% of the electricity generation was accomplished using fossil fuels with only 6.3% contribution from renewable sources including solar energy. A projection by World Energy Council shows that electricity generation needs to be increased to 47 900–53 600 TWh by 2050 to meet the demands of an increased population.^7^ The sun is the most abundant source of energy for the earth with no running cost and leaves no residual waste or pollution to meet this demand. In fact, a theoretical calculation shows that sun provides adequate energy to earth in a day which is sufficient to satisfy the energy needs of all human race for an entire year.^8^ However, a major drawback of solar energy is that it needs to be effectively tapped and efficiently stored, since it is not available throughout a day. Currently, the wearable electronic devices are powered by recharging their battery units after each discharge through a power outlet. This major constraint can be circumvented by integrating energy harvesting and storage parts into a single unit. There are many instances where this is very beneficial for various applications. For example, the military can save considerable human and monitory assets required to deliver power packages to soldiers working in a hostile environment if their jackets are made from fabrics that can harvest and store energy.^9^

The concept of wearable, self‐powered devices has been known for centuries and in fact, a self‐winding watch is one of the earliest self‐powered wearable devices invented in the 15th century. The other examples include calculator watches that entered the market in the 1970s and wearable computers developed in 1970s and 1980s. Presently, technology is in a new phase of developing wearable devices which can continuously monitor human activities and health and sense their environment. Many new wearable devices like electronic skins, artificial muscles, wearable monitors for physiological signals like electrocardiography that are on the horizon.^10^, ^11^, ^12^ For example, if sensors can be embedded into the artificial skin, it can detect toxic gases or chemicals that they are near or in contact with.

An example of the wearable device that entered the commercial market is the Levi's Commuter Trucker jacket with Google's Jacquard garment woven into the clothing.^13^, ^14^ The jacket is the first introduction of smart clothing where touch gestures on the jacket control features on an individual's smart phone through bluetooth connectivity that is embedded in the jacket and paired to an application on the smart phone.

**Figure 1:**
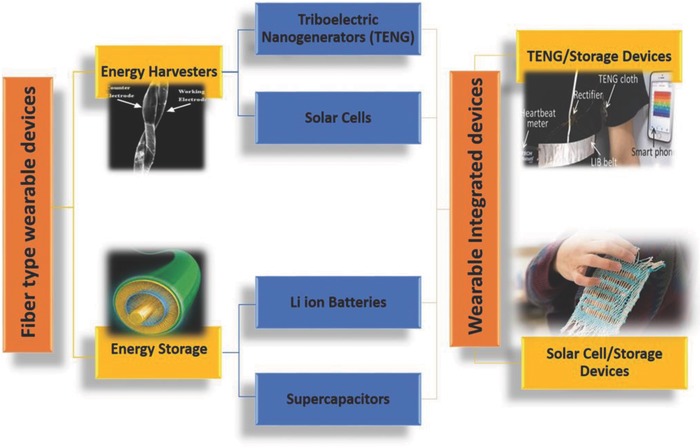
Schematic of the important aspects of this review article. Reproduced with permission.^40^ Copyright 2008, Wiley‐VCH;^222^ Copyright 2015, Wiley‐VCH.

Wearable energy devices make energy portability easy and realistic.^15^, ^16^, ^17^ A fabric or matrix which can simultaneously harvest and store electric energy is the “Holy Grail” for accomplishing easy portability of energy. Some of the recent advances in developing wearable devices are briefly reviewed in the first part of this paper. There are several research efforts in developing independent devices for either energy harvesting or storage given its applications in wearable devices. This includes fiber‐type solar cells, triboelectric generators, batteries, and supercapacitors. Recent advances in this field are reviewed in the second part of the paper. A highly integrated energy harvesting and storing device is very promising for many wearable applications. This eases the need for carrying heavy batteries during important missions including those for first responders and military personnel. A clear direction is the integration of energy harvesting and storing devices on a single platform that is a key to success. However, these devices should be judiciously designed and prudently fabricated to achieve high energy harvesting and storage capabilities. There are a few conceptual designs proposed for developing completely integrated energy harvesting and storage devices.^18^, ^19^ Some of these technologies are reviewed toward the end of this review. A schematic representation summarizing the important aspects of this paper is given in **Figure** **1**.

## 2 Advances in Wearable Devices

The rapid growth of wearable sensors and actuators is evident from a market growth of 25% from 2014 to 2015 with similar growth trends expected in the next five years.^20^ Technologically, such growth is driven by an explosion in sensing and actuation technologies combined with advanced stretchable and conformable materials.^21^, ^22^ This is in conjunction with the commercial strengths of low power silicon electronics and wireless data transmission technologies. In addition to fitness and consumers desire to exercise smarter, such a demand is in part being driven by the desire to monitor vital human signs/responses continuously and conveying this information to medical professionals. This information is used by the medical professionals for establishing baselines for individuals and intervenes in case of abnormalities in these monitored vital signs. In this section, we would like to introduce some of the currently used/proposed wearable devices. Advances in the techniques to power such devices are reviewed in the remainder of the paper.

**Figure 2:**
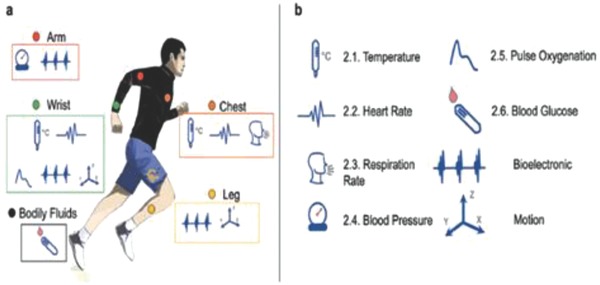
Biosignals and sensing locations: a) the various “wearable” medical devices are indicated in this schematic with locations on the body where they can be noninvasively sensed; b) the various biosignals that can be collected from the various par. Reproduced with permission.^24^ Copyright 2016, Wiley‐VCH.

**Figure 3:**
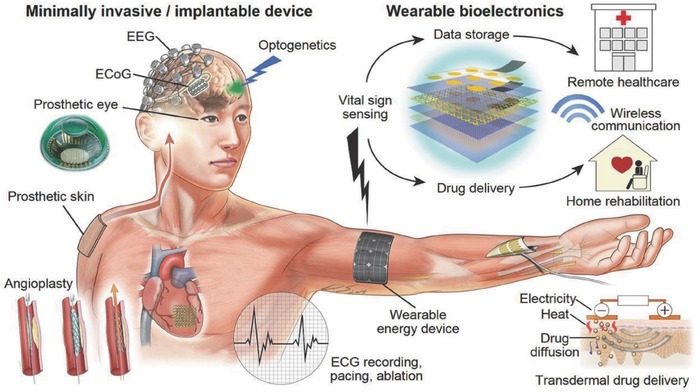
Schematic illustration of various wearable, minimally invasive, and implantable medical devices with the sensing and communication paradigms. Reproduced with permission.^23^ Copyright 2016, Wiley‐VCH.

**Figure 4:**
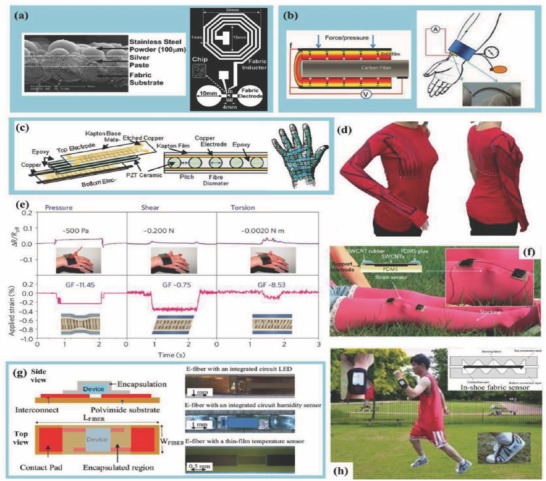
Various fabric and textile based sensors: a) textile based biopotential electrode; b) zinc oxide thin films grown around a carbon fiber acting as a strain sensor; c) vibration sensor arrays for Parkinson's tremor sensing on the hand; d) kinesthetic monitoring with carbon‐loaded elastomeric garment; e) strain gauge sensor with Pt‐coated nanofibers; f) CNT based sensor for human motion detection; g) woven electronic fiber displays; and h) plantar pressure monitoring sensors in the shoes. Reproduced with permission.^25^ Copyright 2014, Wiley‐VCH.

Biofunctional textile is an area that is receiving considerable attention recently. The combination of materials science and micro/nanotechnologies can serve as a major boost and revolutionize some aspects like clothing, sporting goods, food processing, and other markets. Higher impact areas such as military and aerospace have a significant and urgent need for bioinspired and biofunctional textiles with permanent properties such as self‐healing, adaptability, and self‐repair. Some of these devices and textiles are targeted at measuring bodily signatures such as blood pressure, pulse rate, blood glucose, electrocardiography, respiration rate, body temperature, etc. that are available both as fitness monitoring units and as medical devices (**Figures** **2** and **3**).^23^, ^24^ The wearable medical devices as indicated in the schematic (Figure 3) can include invasive devices such as bioelectrode arrays for the measurement of electroencephalography or electromyography.^23^ Similarly, with the development of smart fabrics, the movement from passive to ultrasmart fabrics has begun with integration of sensing and actuation functionalities in fabrics. This transforms textiles from serving the function of “just protection of the body” to truly being able to monitor physiological conditions in an active and continuous manner, as shown in some examples in **Figure** **4**.^25^

Wearable smart textiles incorporate electroactive materials (EAMs) that allow the conversion of mechanical energy to electrical energy and vice versa.^26^ The motion of the body can serve as the mechanical energy required for the energy conversion. The various EAMs that have been utilized include carbon nanotubes (CNTs), conductive polymers, ion polymer–metal composites, ferroelectric polymers, and dielectric elastomers.

As the era of wearable devices is rapidly growing, the demand for lightweight and flexible energy sources that can power these devices has increased considerably.^27^ The powering of these devices by traditional rechargeable batteries dramatically reduces the lifetime of the devices and their sustainable operation.^28^ As a result, there have been significant resources devoted to developing energy sources that can efficiently harvest energy from the environment and store it efficiently to power wearable electronic devices. Energy sources that can be utilized for this purpose include the sun, motion, wind, thermoelectric, electrochemical, triboelectric, etc. The conversion of mechanical energy to electrical energy is common for micro/nanoscale transduction mechanisms such as piezoelectric and triboelectric generators and elastomers.^29^, ^30^ The difference in temperature between the body where the wearable devices reside and the surrounding environment is an ideal source for energy harvesting and extraction for thermoelectric generators. The structural design of the energy harvester and the mechanical and material properties of the energy harvesting materials will control key characteristics of the wearable energy harvesting device. In the following sections, we review some of the important energy sources, both independent energy harvesting and storing devices in addition to integrated devices, which are developed with wearable applications as the end goal. We also discuss the various fiber‐type architectures and materials used for the wearable devices detailing the issues like long‐term durability, stability, failure mechanisms, and performance of these devices in each section devoted to energy harvesting and storing devices.

## 3 Materials and Structure Design

The materials or fiber electrodes developed for the fiber‐based wearable electronics should be fabricated from highly biocompatible and safe materials, which will not impose any threats to the humans or environment. Selecting and designing bendable substrates with soft nature, shape‐conformability, aesthetic diversity, stretchability, and high mechanical durability are some of the key challenges faced in fabricating wearable electronics. These properties can be achieved by replacing existing planar based substrates with fiber ones. These fiber‐based wearable electronics provide the wearer comfort due to the breathability of the fabric. These fiber based devices have good fatigue resistance, as the strain induced during deformations will be affecting only smaller areas unlike crack or peeling‐off of the active material unlike planar substrates. These fibers may need to undergo washing and drying upon integrating into fabrics for thousands of cycles. So a good packaging of the fiber is necessary for a long cycle life. The fiber based energy storage devices are majorly fabricated by the following structural designs as parallel pattern twisted or intertwined pattern and wrapped pattern.

The commonly used fibers for wearable energy devices are made of metals, carbon, CNT, CNT nanocomposites, and graphene. The active material used varies depending on the application. Solar cell utilizes perovskite, organic dyes, or polymers for harvesting solar energy. The triboelectric nanogenerators employ active materials like kapton, polyester, polytetrafluoroethylene (PTFE), polydimethylsiloxane (PDMS), polymethyl methacrylate (PMMA), and polyethylene terephthalate (PET) films for harvesting energy. Lithium ion batteries (LIBs) apply lithium iron phosphate (LiFePO_4_), lithium titanate, lithium cobalt oxide (LiCoO_2_), lithium permanganate (LiMn_2_O_4_) for energy storage. Supercapacitors use materials like carbon, conductive polymers, metal oxide nanoparticles, and their composites for energy storage.

## 4 Fiber‐Shaped Energy Harvesting Devices

It is evident from the previous section that new forms of energy harvesting and storage devices are necessary to power the ever‐increasing demand for wearable applications. In this section, we discuss two types of energy harvesting devices mainly in the form of fibers: i) conversion of light energy into electric energy using solar cells and ii) transforming mechanical energy from a human's daily activities into electrical energy using triboelectric nanogenerators.

### 4.1 Ribbon/Fiber Solar Cells

Recent innovations in the field of energy research have led to the development of stretchable and wearable energy devices in smaller and compact dimensions. Flexible and wearable solar cells play a major role in wearable applications where energy harvesting is highly important. Given weaving the solar cell into a textile, it is ideal to have these solar cells in the form of fiber or ribbons.^31^, ^32^, ^33^ Since inorganic solar cell materials like silicon are not amenable to bending for wearable applications, most of the fiber‐shaped solar (FSS) cells are developed using organic or hybrid materials.

#### 4.1.1 Fiber‐Shaped Dye‐Sensitized Solar Cells (DSSCs)

**Figure 5:**
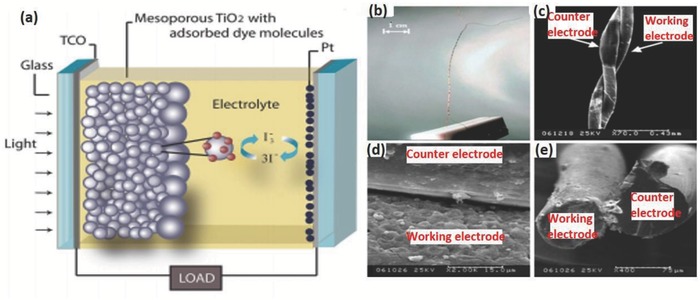
a) Schematic of a DSSC. Reproduced with permission.^34^ Copyright 2012, American Vacuum Society. b) Optical photo of a twisted WSF‐DSSC; c) and d) Scanning electron microscopy (SEM) images of a WSF‐DSSC (top view); e) SEM photo of a WSF‐DSSC (sectional view). Reproduced with permission.^40^ Copyright 2008, Wiley‐VCH.

In DSSCs, the energy conversion is achieved by a photoelectrochemical process that involves light‐induced redox reactions, as depicted in **Figure** **5**a.^34^ DSSC consists of a film of a large bandgap mesoporous semiconducting material like titanium dioxide (TiO_2_) coated with a monolayer of dye molecules like polypyridyl ruthenium dye and filled with a redox electrolyte like I^−^/I_3_^−^. When light falls on the dye molecule, electrons are excited from highest occupied molecular orbital (HOMO) to lowest unoccupied molecular orbital (LUMO). Since its LUMO is above the conduction band edge of TiO_2_, the electron is injected into the TiO_2_ conduction band. The oxidized dye molecule is regenerated by the redox species (by converting I^−^ to I_3_^−^). When the circuit is closed, the electrons migrate to the counter electrode through the external circuit and combine with I_3_^−^ converting it back to I^−^ completing the cycle.

DSSCs offer appreciable power conversion efficiencies (PCEs), low cost, and small incident light angle dependence. The use of fluorine doped tin oxide (FTO) in such devices poses a significant problem to make them flexible and light‐weight devices for wearable applications. Indium tin oxide (ITO) coated polymer substrates can replace the FTO substrates on glass to a certain extent.^20^ However, the efficiencies of these cells are too low compared to the FTO analogs due to their lower conductivities, low heat resistances, and fatigue inherent to the ITO/polymer substrates.^35^, ^36^, ^37^, ^38^ DSSCs in the form of fibers can adequately address most of these issues. The first of this kind of a device was reported in 2001 by Baps et al. in which the working electrode was a steel wire coated with a layer of dye‐absorbed TiO_2_ nanoparticles.^39^ The counter electrode of the tube‐shaped solar cell was a conductive polymer coated inside a transparent polymer tube in which the cell was assembled. The liquid electrolyte was injected in between the wires. The 10 cm long solar cell could deliver a voltage of 0.3–0.35 V and current of only a few µA. However, these liquid electrolytes limit the application of these fibers in wearable devices unless it is completely sealed and well packaged. To avoid the use of transparent conducting oxides (TCOs), a flexible and wearable DSSC fiber was made on twisted fiber‐like electrodes by Fan et al.^40^ The cell used two fiber‐like electrodes: the working electrode was a stainless‐steel fiber coated with a layer of dye‐sensitized TiO_2_, and a conducting Pt wire acted as the counter electrode, twisted in a helical structure (Figure 5b,c). SEM images of the device are given in Figure 5d,e. As the fibers are twisted forming a cell with an apparent diameter of 200 µm, the working electrode received substantial light that can be converted to electrical energy. They also found that the carrier kinetics and adsorption by the dye are greatly influenced by the porous TiO_2_ structure. Such a fiber with 3.5 µm thick porous TiO_2_ layer could give open circuit voltage (*V*_oc_), short circuit current (*I*_sc_), and fill factor (FF) of 0.61 V, 0.06 mA, and 0.38, respectively. It is not clear how the screening effect of the counter electrode affects the efficiency of this fiber DSSC.

Efficient light harvesting is one of the key requirements for enhancing the efficiency of a solar cell. To circumvent the screening effect of the electrodes, it is necessary to develop fiber DSSC bifacial devices. A bifacial, TCO‐free, flexible fiber DSSC can show high efficiency.^41^ They are typically developed using low‐cost metal fibers like steel and titanium as the electrode materials. They harvest solar energy from both sides. Additionally, the use of TiO_2_ nanowire arrays as 1D semiconductors on titanium (Ti) wire enhances the electron transfer rate of the photoanode and improves the flexibility of the fiber‐based DSSCs.^42^, ^43^ These DSSCs could attain PCEs ranging from 0.86% to 5.38%^44^, ^45^ and the efficiency can be improved up to 7%^46^ when the lengths of the TiO_2_ wires are increased to 35 µm on a 250 µm Ti wire. This improvement in PCE is significant for fiber solar cells and may be suitable for some practical applications in wearable technologies if efficiently packaged.

To further improve the efficiency of the fiber‐shaped DSSCs, a TiO_2_ nanocrystal hole blocking layer can be introduced. An efficiency as high as 7.19% was achieved with an 80 µm platinum wire counter electrode.^47^ These cells retained initial PCEs even when the cells were bent. The PCE was further increased to 8.07% by Liu et al. by using a frame of Ti/TiO_2_ microcone array as the electron transfer channel for the photoanode.^48^ Recently, a record PCE of 10% was achieved in a novel design of core–sheath CNT fiber‐shaped DSSC fabricated by twisting hydrophobic and hydrophilic CNT sheets.^49^ The hydrophobic aligned CNT core provides excellent electrical conductivity and mechanical strength, while the hydrophilic aligned CNT sheath helps in effective incorporation of active phases. The fiber‐shaped DSSC had good mechanical stability retaining 82% of PCE after bending at 90° for 2000 cycles. The DSSC was also woven into textile and was used to power a pedometer effectively under sunlight. Owing to the increasing efficiency of these solar cells and compatibility with a variety of designs, fiber DSSCs stand out to be one of the promising candidates for wearable energy harvesters. However, the flexibility of these DSSC fibers is not to the level where they can be incorporated into a wearable device. In addition, the necessity of using liquid electrolyte for DSSC is a major concern for using it in wearable applications.

Apart from the flexible nature, the wearables like artificial skins and various implants in the body experience innumerable stretching actions. Most of the fiber type solar cells detailed above are just bendable and not stretchable. Device design which includes the selection of flexible and bendable components such as the electrodes are the key factors that affect the stability and efficiency of these devices. Buckling or fracture structured metallic films or conducting polymers are used on elastic substrates as the electrodes to achieve ultimate stretchability.^50^, ^51^, ^52^, ^53^, ^54^, ^55^, ^56^ Yang et al.^57^ developed a unique method to develop elastic electrically conducting fibers for their stretchable, wearable photovoltaic devices which maintained a PCE as high as 7.13% under stretching. They used aligned MWNT sheets wound on rubber fibers for the electrodes that gave highly stable and invariable electronic properties under stretching actions. The working electrode was a modified titanium wire twisted over the stretchable and bendable MWNT fibers. A fiber DSSC was made by coating photoactive materials over the Ti wire in a spring form and these are woven on to an elastic fabric that forms the bendable and stretchable wearable device. The same group developed an environmentally adaptive, stretchable, and bendable fiber‐type DSSC that uses a stable, hydrophobic, nonvolatile, and nontoxic gel electrolyte unlike the previous one which uses a liquid electrolyte.^58^ The polymer‐ionic liquid gel electrolyte can withstand temperatures as high as 300 °C and maintains the quasi‐solid state up to 98 °C qualifying the cells for practical applications. Although it exhibited only a PCE of about 5.47% against the 5.99% exhibited by a cell with the same configuration that uses a liquid electrolyte, the device gives a promising future for the commercialization of highly elastic wearable devices.

#### 4.1.2 Fiber‐Shaped Polymer Solar Cells

**Figure 6:**
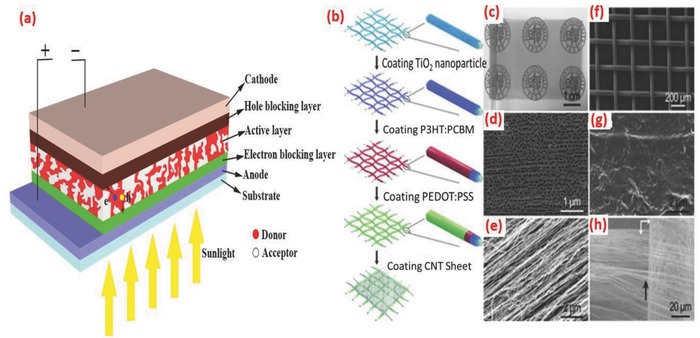
a) Schematic of a typical BHJ polymer solar cell. b) Fabrication of the PSC textile. c) Photograph of a Ti textile with one part wrapped around a glass rod and the other part on a labeled paper. d) SEM image of Ti textile. e) Aligned TiO_2_ nanotubes. f) Layers that were coated with P3HT:PCBM and PEDOT:PSS. g) Aligned CNT sheet. h) Aligned CNT sheet closely attached to the surface of the Ti textile. Reproduced with permission.^62^ Copyright 2014, Wiley‐VCH.

Polymer solar cells (PSC) use the polymer as the active materials for light harvesting. PSC is an attractive choice for FSS cells because of its flexibility, lightweight, and low‐cost fabrication to achieve the solar cell structure. A typical solar cell consists of the following components: donor (D) molecules, acceptor (A) molecules, electron blocking layer (EBL), hole blocking layer (HBL), hole collecting electrode (HCE), and electron collecting electrode (ECE), as shown in **Figure** **6**a. The most commonly developed PSC is a bulk heterojunction (BHJ) solar cell in which electron donor and acceptor materials are intimately mixed and cast as a single layer between EBL on HCE and HBL on ECE.^59^ When the cell is illuminated, electron–hole pair (exciton) is generated at the BHJ layer. At the D–A interface, due to the difference in the chemical potential, the exciton dissociation takes place and the electron is transferred to the LUMO of the acceptor and the hole to the HOMO of the donor molecule. The electron moves to the ECE through the HBL and hole to the HCE through EBL to generate an open circuit voltage (*V*_oc_). When the circuit is closed, the electron moves through the external load to the HCE to complete the circuit.

The symmetry, tunable aspect ratio, and linear structure of fibers could be utilized in FSS cells for effectively harvesting energy. Although the fiber‐shaped DSSCs could deliver efficiencies up to 8%,^48^ they are not ideal for wearable application, since they require additional packaging due to the presence of volatile electrolytes. Solid inorganic or organic FSSs overcome this disadvantage even though the efficiency is sacrificed to a certain extent.^33^, ^60^, ^61^ A major issue with solid fiber‐shaped photovoltaic cells is the balance between optimum transparency and conductivity of the top electrode. For developing a high performing FSS, a high transparency for efficient light harvesting and a good electrical conductivity for efficient charge collection are necessary. Flexible and lightweight PSC textiles developed by Zhang et al. reported a similar PCE irrespective of the side from which it is illuminated.^62^ They were fabricated by dip coating poly(3‐hexylthiophene):phenyl‐C61‐butyric acid methyl ester (P3HT:PCBM) as photoactive layer on a textile made from micrometer‐sized Ti metal wire (cathode) with perpendicularly aligned TiO_2_ nanotubes (Figure 6b–e). CNT sheets with appreciable mechanical and electrical properties were used as the anode (Figure 6f). A photograph of the fiber developed is shown in Figure 6g and a schematic of the fabrication procedure stretchable fiber‐shaped PSC is given in Figure 6h.

**Figure 7:**
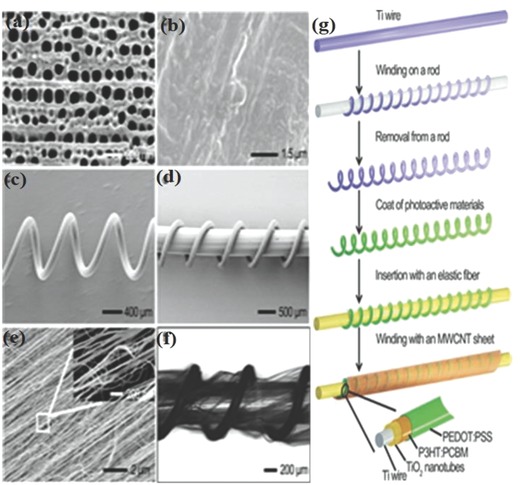
Structural characterization of the stretchable fiber‐shaped PSC. a) Perpendicularly aligned TiO_2_ nanotubes, top view. b) PEDOT:PSS layer on the P3HT:PCBM layer that was coated on the TiO_2_ nanotube array, top view. SEM image of c) the spring‐like modified Ti wire after coating of the PEDOT:PSS layer. d) Spring‐like Ti wire inserted with an elastic fiber. e) MWCNT sheet (inset: high magnification). f) Photograph of a fiber‐shaped PSC. g) Schematic illustration of the fabrication of a stretchable fiber‐shaped PSC. Reproduced with permission.^63^ Copyright 2015, Wiley‐VCH.

Although the efficiencies of all these solid‐state fiber solar cells were low, the PCEs were stable even after 200 bending cycles. For practical applications like electronic textiles, flexibility and stretchability are very important. Many of the current planar photovoltaic devices and FSS cells are not bendable and stretchable and thus incapable of handling stress due to movements and are therefore prone to damages.^34^ To achieve bendable and stretchable FSS cells, a spring‐like elastic substrate is used by Zhang et al.^63^ In this architecture, a Ti wire was wound around a rod to acquire a spring‐like shape (**Figure** **7**). Aligned TiO_2_ nanotubes were grown by an electrochemical method on the Ti wire, and a photoactive layer (P3HT:PCBM) was coated on top of the wire. Subsequently, a hole transport layer poly(3,4‐ethylenedioxythiophene):poly(styrenesulfonate) (PEDOT:PSS) was coated on the photoactive layer and a flexible and bendable rod made of rubber was inserted into the Ti spring. A 18 nm thin layer of multiwalled carbon nanotube (MWCNT) sheet was subsequently wound over the fiber as the second electrode. The PCEs of these FSSs were found to maintain 90% of the initial value even after 1000 cycles of bending, unlike the conventional planar polymer solar cells. A major limitation of the spring‐like architectures for the FSS cell is that a major part of the total area of the solar cell is not exposed to the sunlight. Therefore, the total power output of the spring‐like solar cell is inferior to a typical FSS.

#### 4.1.3 Fiber‐Shaped Perovskite Solar Cells

**Figure 8:**
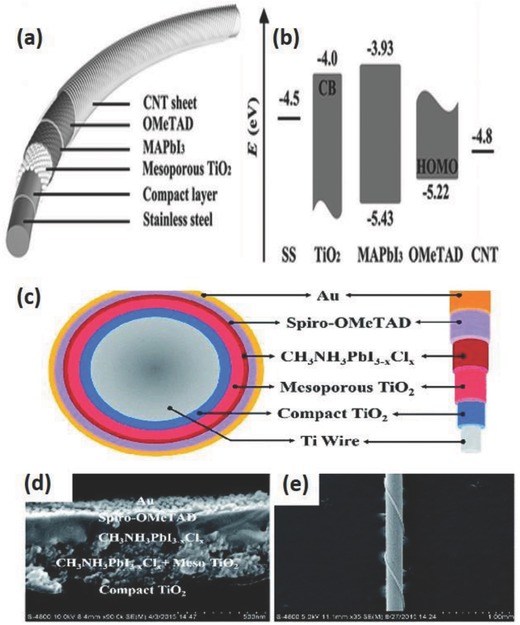
a) Structure and b) energy‐level diagram of the fiber‐shaped perovskite solar cell. Reproduced with permission.^67^ Copyright 2014, Wiley‐VCH. c) Schematic of the fiber‐shaped perovskite solar cell (FPESC) structure. d) SEM cross‐sectional image of the FPSC. e) Image of typical FPSC. Reproduced with permission.^69^ Copyright 2016, RSC Publishing.

Currently, perovskite solar cells (PESC) with organolead halide perovskites are getting considerable attention due to their high efficiencies (about 22%)^64^, ^65^, ^66^ and ease of fabrication. The fiber‐shaped perovskite solar cell (FPESC) also works similar to PSC. The basic functioning of an FPESC is shown in **Figure** **8**a and the band diagram is illustrated in Figure 8b. Briefly, the excitons are generated in the perovskite layer because of illumination. These excitons dissociate into electrons and holes and the electrons are transferred to the electron transport layer (ETL) and holes to the hole transport layer (HTL) from the perovskite layer. Following this, the carriers are transferred to the respective electrodes like in PSC.

Typically, a PESC is built on a flexible metal wire like stainless steel or titanium which acts as the cathode.^67^ A compact layer and a mesoporous layer of titanium dioxide (TiO_2_) are deposited as HBL and ETL, respectively, before making a perovskite layer around the fiber. This is followed by the deposition of a HTL like spiro‐OMeTAD [2,2′,7,7′‐tetra‐kis(N,N‐di‐para‐methoxyphenyl‐amine)‐9,9‐spirobifluorene]. A transparent conductive material like the CNT sheet or a very thin layer of gold is wrapped around this structure function as the anode. As the light is absorbed by the perovskite layer, the excited charge carriers are effectively separated to the HTL and ETL to constitute the photovoltaic current.

Owing to the high charge generation efficiency of perovskite materials and the feasibility to be wet processed, they are a good candidate for making FSS. Another practical approach to make FPESC is sandwiching a perovskite layer between a fiber electrode and another semi‐transparent film electrode wrapped around it. Qiu et al. developed such a solar cell using semi‐transparent MWCNT sheet electrode continuously wound over the inner fiber with the perovskite layer.^67^ The all‐solid‐state FPESC provided a PCE of 3.3% even after 50 bending cycles. The photovoltaic parameters like the *V*_oc_, *I*_sc_, and FF remain unchanged even when the angle of illumination was varied from 0°–180° due to the symmetric coaxial architecture.

Retaining the PCE with repeated bending and stretching actions is one of the main aspects to be addressed while developing the fiber solar cells for wearable applications. A double‐twisted FPESC can be very beneficial for making flexible wearable devices. Such an architecture was followed by Li et al. by using CNT electrodes.^68^ The coaxial fiber shaped cells packed in a transparent polymer film provided a bending stability for more than 1000 cycles and a PCE of 3.03%. Moreover, it retained a PCE of 89% even after 96 h of illumination under ambient conditions. A FPESC was fabricated by following an easy‐to‐fabricate and energy saving architecture with Ti/c‐TiO_2_/meso‐TiO_2_/perovskite/spiro‐OMeTAD/Au, as shown in Figure 8c–e.^69^ The cells exhibited an efficiency of 5.35% under AM 1.5 illumination and an apparent efficiency of 8.4% in a diffuse model. Gold nanoparticles were used as the semi‐transparent top‐electrode to avoid the use of any TCOs. The elimination of TCOs is advantageous to achieve flexibility for use in wearable applications. However, c‐TiO_2_ compact layer affects the flexibility of the FPESC. A bending test to study the degradation of these devices is important to learn its application in wearable devices.

Another interesting approach to make efficient FPESC is a cathodic deposition technique.^70^ The advantage of this approach is that a uniform layer of perovskite can be obtained on curved surfaces. A compact TiO_2_ layer is prepared on Ti wire as an electron transport layer. A TiO_2_ nanorod array can be radially grown through an anodization process. This nanorod array behaves as a mesoporous surface for the extraction and transport of electrons. The anodization voltage was varied from 10–20 V to achieve nanorods with diameters ranging from 45–100 nm. A sponge‐like lead oxide (PbO) is prepared on TiO_2_ nanorod array that is converted to lead iodide (PbI_2_) by reacting with hydroiodic (HI) acid. The perovskite layer can be grown by simply treating it with methylammonium iodide (CH_3_NH_3_I). The second electrode, a thin film of CNT with more than 80% linear transmittance in the visible spectrum, was made on the perovskite layer by spin coating an array of aligned CNTs with isopropanol. These FPESCs exhibited a high *V*_oc_ of 0.85 V and a PCE of 7.1%. This method to develop PESC on curved surface is very useful as an energy source for wearable devices, since oftentimes wearable devices need to be conformal to the body. Bendability remained a problem in FPESC for practical applications until Deng et al. came out with their elastic perovskite solar cell which uses a modified spring‐like Ti wire and a stretchable aligned CNT based fiber as the two electrodes.^71^ These fiber‐type cells were claimed to achieve energy conversion efficiencies ranging from 4.81% to 5.22%. Even after 250 stretching cycles, the devices retained 90% energy conversion efficiencies that make these elastic cells another feasible candidate for practical applications.

In a very recent approach, a non‐halide lead source, lead acetate was used for the first time by Hu et al. toward developing FPESC with large grain size and uniform pinhole‐free perovskite films.^72^ Attaining such a morphology minimizes the defects in the perovskite film, which has the potential to suppress the interface charge recombination. Thus, the FPESC developed with the non‐halide lead source could obtain a high *V*_OC_ of 0.96 V and PCE of 7.53%. FPESC provides high PCE and uses no liquid electrolytes. Therefore, the packaging of FPESC is much easier than DSSC. In addition, properties such as high bending stability, appreciable surface coverage, high efficiency, and leak‐proof nature make perovskite PSSs a promising candidate for the future wearable applications. However, toxicity of lead perovskite is a concern. Serious efforts to replace lead with other non‐toxic materials are currently in progress.^73^, ^74^, ^75^, ^76^ Long term stability is the most important issue that impedes the perovskite based solar cells from rapid commercialization. The environmental and thermal stability of these materials can be improved by several methods like using modified perovskite structures which replaces the organic cation with cesium and rubidium.^77^, ^78^, ^79^ These modifications can not only improve the overall stability of the solar cell but also enhance the efficiency to a great extent.

### 4.2 Fiber‐Based Triboelectric Nanogenerators

**Figure 9:**
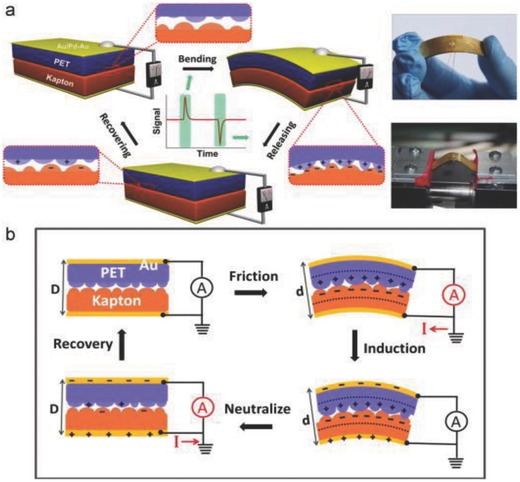
Schematic illustration of the structure and working principle of the triboelectric generator. a) The structure of an integrated generator in bending and releasing process and related electrical measurement tests. Photographic images of a flexible TEG and mechanical bending equipment. b) Proposed mechanism of a TEG. Reproduced with permission.^92^ Copyright 2012, Elsevier.

The overwhelming demand for self‐powered wearable nanodevices and nanomachines has initiated the development of compact and flexible energy harvesters and generators from renewable sources. There are various renewable energy sources other than solar and wind energies; like the piezoelectric,^80^, ^81^, ^82^, ^83^, ^84^, ^85^ pyroelectric,^83^, ^86^, ^87^, ^88^ and electromagnetic effects.^89^, ^90^, ^91^ The triboelectric process is one such kind associated with our daily activities that involves the relative motion between two surfaces that can generate considerable energy that is currently wasted. This mechanical motion converted to electrical energy is adequate to power many micro‐electro‐mechanical systems and other similar devices. Such a device that works by the combined effect of contact electrification and electromagnetic induction is called a triboelectric nanogenerator (TENG).^29^, ^92^ The nature and strength of the charges depend upon the strain, surface roughness, temperature, and type of materials. The first triboelectric nanogenerator was introduced by Fan et al. at Georgia Tech in the year 2012.^92^ In this device, the charge generation, separation, and induction processes were achieved by the mechanical deformations of two thin polymer films of Kapton and polyester (PET) of dissimilar tribopolarity sandwiched together (**Figure** **9**a). Contacts from either side of the stacked device were obtained using a layer of 100 nm thin film of gold. The metal film also serves to produce equal and opposite charges from the potential derived from the triboelectric process due to electromagnetic induction. The proposed mechanism of TENG is illustrated in Figure 9b. Mechanical compression (reduction in interplanar distance between electrodes from D to d) causing friction between the two polymer films generates electrostatic charges of opposite signs, which in turn develops an interface dipole layer called as triboelectric potential layer. The insulating behavior of the polymer films slows down the conduction or neutralization of induced charges. Under the driving force of tribolelectric potential, change in system capacitance leads to current flow across the external load to minimize the energy created by triboelectric potential. The scalable, flexible, polymer‐based, eco‐friendly, and low‐cost nanogenerator developed delivers a peak power density of 10.4 mW cm^−3^ at a peak voltage of 3.3 V at 0.6 µA. Within a year, the output power density of the TENG increased to approximately five orders of magnitude and the conversion efficiencies to 60–72.4%.^29^ A considerable volume of papers has been published since its inception in 2012.^26^, ^29^, ^93^, ^94^, ^95^, ^96^, ^97^, ^98^, ^99^ Recently, fiber type TENGs have been reported with flexible, soft, breathable, and wearable characteristics suitable for energy generation and integration into textiles.^26^, ^100^, ^101^

**Figure 10:**
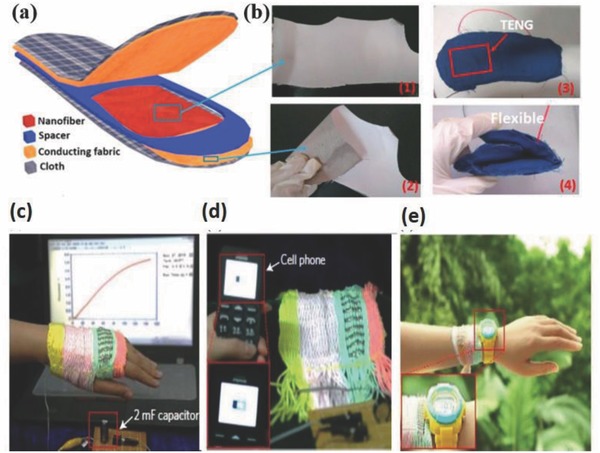
a) Schematic diagram of the structure of the TENG‐based insole. b) Digital photographs of the as‐spun PVDF nanofibers on the conducting fabric 1) front side and 2) back side, 3) and 4) are photographs of the fabricated TENG‐based insole demonstrating its flexibility. Reproduced with permission.^99^ Copyright 2015, Elsevier. Demonstration of the power textile to drive portable electronics. c) Under natural daylight with mechanical excitation, charging a 2 mF commercial capacitor up to 2 V in 1 min, d) directly charging a cell phone, and e) continuously powering an electronic watch in a wearable manner. Reproduced with permission.^103^ Copyright 2016, Springer Nature.

An attractive approach to make high‐performing wearable TENG is using fibers made by electrospinning. The highly porous electrospun fibers so prepared can not only replace the use of dense films which are not completely air permeable but also offer a larger contact area that enhances the output power by improved friction between the surfaces. Recently, Huang et al. fabricated the first wearable, substrate‐free, all‐fiber insole based TENG (**Figure** **10**a) by an electrospinning method that could produce a voltage of 210 V, 45 µA current, and 2.1 nW output power.^99^ They replaced the typically used negative triboelectric materials such as PTFE and PDMS with the polyvinylidene fluoride (PVDF), a strongly electronegative material with better suitability to act as fabric for wearables (Figure 10). The positive triboelectric PMMA and PET were replaced with copper and nickel‐coated PET which eliminates the necessity of an extra metallic film for charge collection. Figure 10b(1),(2) are photographs of the as‐spun PVDF nanofibers, which were firmly integrated with the conducting fabrics. The flexibility of the fabricated TENG‐based insole is demonstrated in Figure 10b(3),(4), which ensured its durability and wearability.

A highly flexible, sensitive, stable, and lightweight, coaxial fiber‐based TENG (FTENG) can be very advantageous for energy scavenging and sensing. The FTENGs use porous PDMS and PMMA as the triboelectric materials with aligned CNTs as the inner and outer electrodes. The devices are capable of converting mechanical energies from any stimuli arising from bending, stretching, pressing, twisting, and vibration. The FTENG is tested to sense motion of finger joints and detect the direction and velocity of moving objects.^102^ This study reveals the possibility of using these devices in flexible and wearable intelligent electronics for velocity detection, personal health‐care, and tracking the traffic.

Energy generated by triboelectric nanogenerators is typically low. To improve the energy output, Chen et al. developed a wearable, lightweight, foldable, and flexible, microcable power textile which can simultaneously gather energy from sunlight and mechanical movements.^103^ Solar cells fabricated from polymer fibers in the form of microcables were woven into fiber‐type TENGs using a “shuttle‐flying” process to form the smart textile (Figure 10). The thickness of such a single layer fabric is about 320 µm and can be easily integrated into any textile of interest. A commercial capacitor of 2 mF was charged to 2 V in 1 min when such a fabric of dimensions 4 cm × 5 cm was exposed to ambient sunlight in the presence of mechanical stimuli like blowing wind or human movements (Figure 10c). It is feasible to use this textile to continuously power a watch (Figure 10d), a cell phone (Figure 10e), and drive water‐splitting reactions thereby demonstrating the feasibility and scope for high power applications.

For wearable applications especially in the form of textiles, air permeation is very important. Corrugated structures that can be easily stretched are desirable. A corrugated textile using TENG could not only generate energy by pressing and rubbing, but also by stretching actions.^26^ The corrugated structure has the advantage of providing the required air gaps without the use of extra spacers, and it delivers a peak voltage of 28.13 V and 2.71 µA by simple “stretch and release” actions. Further, the suitability of this kind of architecture for self‐powered systems was successfully demonstrated by powering 54 light emitting diodes from various human movements.

**Figure 11:**
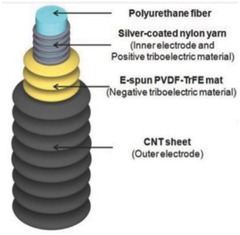
Schematic diagram of stretchable triboelectric structure. Reproduced with permission.^104^ Copyright 2016, Springer Nature.

A high cycling performance is indispensable for wearable TENG devices. A stretchable and weavable fiber and yarn TENG of a few micrometers diameter with high cycling performance was developed by Sim et al.^104^ In this TENG, silver‐coated nylon/polyurethane was the inner core fiber, and a layer of wrinkled polyvinylidene fluoride‐co‐trifluoroethylene/CNT was used as the shell, as shown in **Figure** **11**. The tensile change of these materials provides the observed electrical output. The dependence of electrical output with applied strain of a 3 mm fiber was evaluated by fixing one end of it on a clamp and the other end on a motor operating at a frequency of 10 Hz that is comparable to usual human motion. The voltage and current varied from 13 to 24 mV and 3 to 8 nA, respectively, when the strain was increased from 10% to 50% indicating an appreciable performance of the stretchable fiber. This structure could retain remarkable performance with stable voltage response even after 10 000 cycles of stretching and releasing actions. Hence, it can be effectively used on human body parts like the fingers, elbows, and knees that undergo high deformation. These fibers were woven into the commercial fabric for textile‐based sensors and tested successfully to determine the magnitude and direction of motion.

In general, FTENGs are an important energy generating device candidate for wearable energy devices. The basic idea of harvesting energy from body movements is highly advantageous for wearable energy devices. Additionally, their high peak voltage is beneficial for charging many electronic devices. However, the low power output of TENG is a serious constraint for its implementation in wearable devices. Coupling TENG with a solar cell is an interesting direction to pursue.

## 5 Fiber‐Shaped Energy Storage Devices

**Figure 12:**
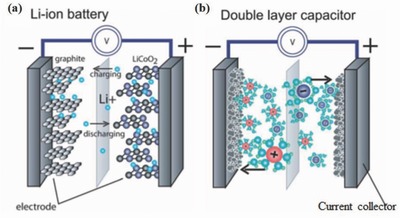
Basic schematic showing a) Li‐ion battery and b) double layer supercapacitor. Reproduced with permission.^105^ Copyright 2014, RSC Publishing.

Harvested energy has to be stored efficiently in energy storage devices for further applications. Generally, LIB and supercapacitors are used for this purpose. Typical charge storage mechanism of intercalation–deintercalation (ions stored in between layers of electrode materials) and double layer capacitance (ions stored on the surface of electrode material) in LIB and supercapacitors, respectively, are shown in **Figure** **12**a,b.^105^ This section will focus on wearable fiber shaped and stretchable LIB and supercapacitor.

### 5.1 Wearable LIB Fibers

Next generation electronic devices require portable power sources for charging them. Considering applications, such as roll‐up displays, touch screens, wearable sensors and implantable medical devices, flexible and wearable power sources, which are highly efficient is indispensable for such applications.^105^ Among all energy storage devices, LIB has revolutionized the modern consumer electronics industry owing to its high energy density, light‐weight, good cyclic stability, and environmentally friendly operation. Recently, many researchers are working toward the development of thin and flexible LIBs.^106^, ^107^, ^108^ In developing wearable devices, a fiber‐shaped LIB offers considerable advantages over a planar structure, since it can be easily bent, twisted, stretched, and woven into porous textiles that allow air permeation. The fiber‐shaped devices are important for wearable applications, since the fabric developed can easily adapt to any surface. This section focuses on fiber and cable type LIBs developed for applications in wearable devices.

Typically, LIB consists of an anode and cathode immersed in an electrolyte separated by an ion‐permeable membrane.^105^ The cathode is usually made up of materials like LiCoO_2_, LiMn_2_O_4_ (LMO), or LiFePO_4_ (LFP). Carbon‐based materials such as activated carbon (AC), CNT, or graphene are used as anodes. These active materials are deposited on a conductive metal like copper as the charge collector electrode to aid transferring of charges.^109^, ^110^ The electrolyte is prepared by dissolving a Li salt in an organic solvent. The Li‐ion battery delivers power by the deintercalation of the Li^+^ ions from the anode and transferring it to the cathode through the electrolyte. During this process electrons released from the anode flow to the cathode through the external circuit. At the cathode, the Li‐ions will be intercalated into the electrode along with electrons. The process is reversed during charging.

As mentioned above, for the development of wearable LIBs, appropriate current collector electrodes deposited with respective active electrode materials are required. These components determine the shape and dimensions of the LIB. Since LIB contains liquid electrolytes, it should be completely protected when fabricated in the form of fibers for wearable energy storage applications. The active material for the Li‐ion intercalation should be strongly attached to the conductive fiber electrode by physical or chemical methods.^111^, ^112^, ^113^ In addition, as the wearable devices undergo vigorous mechanical motion, all the components should be supportive to endure the mechanical stresses. Therefore, the fiber electrodes should be (a) highly conductive to ensure rapid charge transfer, and (b) highly flexible and bendable to endure weaving and other stresses during their integration and service time. To accomplish this goal, CNT based fiber electrodes were recently developed.^114^, ^115^, ^116^ The CNTs spun into fibers satisfy the requirements such as high electrical conductivity, tensile strength, flexibility, and light‐weight for fiber LIBs (FLIBs). In addition, the aligned CNT electrodes made by dry spinning can work as a current collector electrode to which active material can be easily deposited without the need for an additional binder.^114^

**Figure 13:**
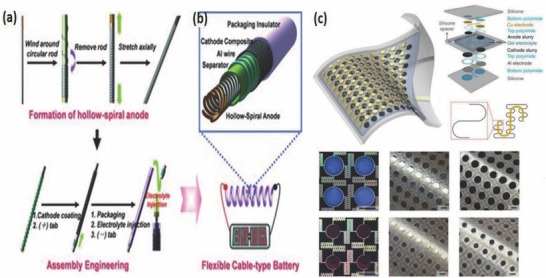
a) Schematic diagram showing fabrication of the cable battery. b) Schematic illustration of the cable battery with hollow‐helix anode having multiple‐helix structure. Reproduced with permission.^117^ Copyright 2012, Wiley‐VCH. c) Aspects in battery layout and design. Reproduced with permission.^51^ Copyright 2013, Springer Nature.

In general, there are two geometrical designs for FLIBs, helically coaxial and twisted/parallel. Recently, Kim and co‐workers^117^ developed a helically coaxial LIB with hollow spiral multihelix anode structure that was vital for good mechanical stability and flexibility. Copper wires deposited with nickel (Ni)–tin (Sn) active materials were twisted to form a bundle of four such wires integrated into a hollow structure anode and PET is used as the separator and aluminum wire as cathode current collector (**Figure** **13**a). A heat shrinkable tube was used to assemble electrodes (Figure 13b). These FLIBs delivered a voltage of 3.5 V and a capacity of 1 mA h cm^−1^. The hollow anode facilitates the electrolyte accessibility and wettability to all components of powering a light‐emitting diode (LED) screen under deformation and bending conditions. To make a coaxial light‐weight fiber for wearable applications, one can replace the metal electrode with aligned CNT/Silicon (Si) fiber as the anode and CNT/LMO as the cathode.^118^ The fiber electrodes developed were wound around a cotton fiber with a layer of gel electrolyte in between. The electrode is subsequently inserted into a shrinkable tube that protects the fiber. It was found that CNT based fiber can provide an initial specific capacity of 106.5 mA h g^−1^ and a voltage output of 3.4 V. Even when Si with high theoretical capacity was used as the active material along with CNT in the above devices, a high electrochemical capacity was not obtained because of the fragile nature of deposited Si. A FLIB with a parallel structure utilizing a poly (vinylidene fluoride) membrane separator was developed using a CNT/LMO cathode and a CNT/Li_4_Ti_4_O_12_ (LTO) anode.^108^ It was packaged into a heat‐shrinkable tube, and the device developed was highly flexible and bendable. An attractive feature observed in these fiber batteries is that the spinel structure of LTO provided zero‐strain for Li‐ion intercalation in addition to higher lithiation potential (1.5 V vs Li/Li^+^) preventing the dendritic growth on the anode. The parallel FLIB provided an energy density of 17.7 W h L^−1^ and a power density of 560 W L^−1^. Preventing Li dendritic growth and achieving reasonably good performance are very positive aspects of this design for wearable applications.

To improve the mechanical endurance of the battery, a wearable textile battery with Ni‐coated polyester yarn as the current collector, LTO and LFP as the active electrodes, and a polyurethane (PU) film separator can be used. PU can also be used as the binder material. The advantage of using this design is that the assembled full cell can show excellent endurance for severe mechanical tests while delivering comparable electrochemical properties.^119^ The presence of urethane groups and polyethylene glycol/polytetramethylene ether glycol (PEG/PTMEG) offer good wettability for both the electrodes. The hard domains of the PU separator provide the mechanical strength, and the soft domains provide flexibility for the yarn battery. Both the binder and the separator provide efficient Li‐ion diffusion through interaction with the polar electrolyte because of the presence of hard domains of urethane groups and the PEG/PTMEG units in the soft domains. The tolerance of the textile battery can be compared with the Al foil based battery over repeated folding and unfolding tests. These tests can provide the robustness of textile battery under mechanical stress. Even during charging–discharging cycles, the textile battery can exhibit excellent electrochemical stability.

In many wearable devices, tensile deformation is an important requirement. To achieve tensile deformation of the whole device, the stretchability of the energy storage devices is inevitable. For example, a wearable device such as an epidermal sensor requires a stretchable energy device to power the sensing functionalities. Xu et al. fabricated a stretchable, rechargeable battery on a silicone elastomer with segmented layouts and iterative interconnection in similar geometries.^51^ The structure of the battery is shown in the Figure 13c. The current collectors for cathode LiCoO_2_ (LCO) and anode LTO are aluminum and copper disks, respectively. The electrode units are connected in a serpentine geometry with gel electrolyte that protects the device from biaxial stretch deformations. The parallel configuration of the device provided an output voltage of 2.5 V. The device can be stretched up to 300% of its original dimensions without degradation. The soft and compliant nature of the device makes it compatible with the biological epidermis.

A suspension–deposition scrolling technique can also be used to fabricate FLIB. In this design, CNT/LTO and CNT/LMO can be used as cathode and anode, respectively. The anode and cathode fibers can be wound on a PDMS elastic matrix with a layer of the gel electrolyte. The stretchability of the fiber can be achieved by applying a layer of PDMS on the surface. A fiber so developed provided a specific capacity of 91.3 mA h g^−1^ with ≈93% of capacity retention even after stretching the device to ≈200%.^120^ Moreover, an impressive 88% capacity retention was achieved even after stretching the device 600% that is very useful for wearable applications. Zhang et al. provided a twist to the traditional stretchable LIB by developing a spring‐like FLIB using CNTs.^121^ This is achieved by increased twisting of several CNT/LMO fibers used as the positive electrode and CNT/LTO hybrid fiber as the negative electrode with a layer of gel electrolyte in between. These two electrodes are twisted and encapsulated in a heat shrinkable tube. The spring‐like structure provided the stretchability with the best deformation of ≈300%. The specific capacity obtained was 92.4 mA h g^−1^. A capacity retention of 92.1% was demonstrated even after 100 cycles. Additionally, the device showed less than 1% capacity fade rate in a stretching test for 300 cycles with less than 50% strain. The twisting preparation method ensures that the fibers are lightweight for applications in the wearable devices space. Very recently, scalable, economical, 3D printing technology was adapted to develop a flexible all‐fiber LIB.^122^ Ink for the printing was made by mixing CNTs with LFP and LTO for cathode and anode, respectively. After 3D printing of the electrode material, the fibers were twisted together with gel polymer electrolyte to form an all‐fiber solid state energy storage device. The all‐fiber LIB exhibited a high specific capacity of 110 mA h g^−1^, maintaining a good flexibility and stability. Their ability to light up an LED even in the bending state indicates the possibility of incorporating the all‐fiber LIB into wearable devices. Moreover, its all solid‐state components allow them to be woven into fabric.

### 5.2 Supercapacitor Fibers

Owing to the nontoxic aqueous electrolytes used, and tens of thousands of cycle life,^123^, ^124^, ^125^ supercapacitors are an excellent choice for energy storage for wearable devices. Like batteries, supercapacitor consists of an anode, cathode, and a separator, but energy storage mechanism is somewhat different. Unlike batteries, supercapacitors store energy on the surface of the electrodes by an electrochemical double layer (EDLC) or pseudocapacitive mechanism. Since the energy is stored on the surface of the electrode, the surface area of the electrodes is critical to enhancing the capacitance of the supercapacitors. They can provide high power densities, long cycle life, and rapid charging–discharging rates over a wide range of operating temperatures. Introducing new configurations for supercapacitors has become crucial to comply with the modern energy storage markets for portable and wearable electronic devices. Based on the applications, special configurations like yarn based,^126^, ^127^ cable type,^128^, ^129^ and screen‐printed supercapacitor^130^, ^131^, ^132^ have been developed. Such configurations provided flexible, lightweight, shape conformable, and mobile usable characteristics for supercapacitors.

#### 5.2.1 Flexible Yarn Supercapacitors

Flexible yarn supercapacitors have linear or 1D architecture and offer several advantages over supercapacitors with planar or 2D architecture and 3D forms.^133^, ^134^, ^135^, ^136^ Planar supercapacitors usually have their rear electrode fabricated from metal sheets, foams, papers, or textile substrates.^105^, ^137^, ^138^, ^139^ In the case of a planar architecture, presence of large area collector electrodes, active materials, conductive additives, and separator membranes results in devices without adequate flexibility, bendability, and air permeability that are necessary for wearable devices.^111^, ^140^ The supercapacitors with linear architecture made from fiber electrodes have an interlaced structure, which allows the constituent filaments to move freely relative to each other providing freedom for body movements and permeability to air and moisture.^141^ These linear architecture devices are fabricated on linear substrates such as metal wires, plastic/rubber wires, carbon wires, carbon nanofibers, CNT yarns, CNT nanocomposites fibers, graphene fibers, and graphene composite fibers.^129^, ^142^, ^143^, ^144^, ^145^, ^146^, ^147^, ^148^ The substrate serves as both the support structure and the current collector for the active materials. Typically used active materials are carbon nanoparticles, metal oxides, and conducting polymers.^144^, ^149^, ^150^, ^151^ Several device architectures have been developed for yarn supercapacitor, such as biscrolled or two‐ply yarn, multi‐ply yarn, braided, core–shell, and coaxial yarns.^134^, ^152^, ^153^, ^154^, ^155^, ^156^

**Figure 14:**
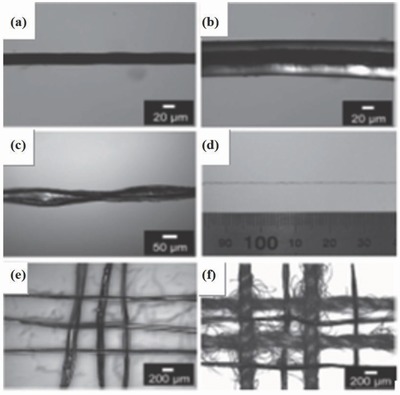
Optical microphotographs. a) CNT@PANI yarn; b) CNT@PANI@PVA yarn; c) two CNT@PANI@PVA single yarns twisted together to form a thread‐like, two‐ply yarn supercapacitor. d) Photograph of a longer supercapacitor. e) A model woven energy storage device consisting of six two‐ply yarn supercapacitors (reflection mode). f) Yarn supercapacitors are cowoven with conventional cotton yarns to form a flexible electronic fabric with self‐sufficient power source. Reproduced with permission.^157^ Copyright 2013, Wiley‐VCH.

Two‐ply yarn supercapacitors, as the name indicates, are fabricated using twisting two strands of fibers of which one acts as the anode and the other acts as the cathode. Both symmetric and asymmetric configurations can be developed for fiber supercapacitors. Wang et al. developed a two‐ply yarn supercapacitor by twisting a continuous CNT web drawn from solid state MWCNT forest and ordered polyaniline (PANI) nanowire of 50 nm in diameter and 400 nm in length (CNT@PANI yarn).^157^ As a high capacitance electrode material, PANI maximizes the performance of the thread‐like supercapacitor. Polyvinyl alcohol (PVA) gel electrolyte was coated on the surface that acts as both a separator and an electrolyte making the CNT@PANI@PVA composite, as shown in the **Figure** **14**. The symmetric arrangement was fabricated where two similar CNT@PANI@PVA yarns were twisted together to form the two‐ply yarn supercapacitor. The thickness of the PVA gel in the yarn supercapacitors is extremely important, as a reduced thickness can cause a short circuit while higher thicknesses might be a problem for weaving and knitting processes. In this work, the authors controlled the thickness of the solid electrolyte to about 20 µm to get a yarn fiber (CNT@PANI@PVA) of ≈60 µm. The fiber was constructed by twisting two yarn electrodes with a total diameter of about 120 µm. These yarns are thinner than the commonly used cotton yarns in lightweight fabrics. The optical micrograph of a fabric model composed of four conventional two‐ply cotton yarns and four two‐ply CNT@PANI@PVA yarn is shown in Figure 14. The structure provided an aerial capacitance of 38 mF cm^−2^ at 0.01 mA cm^−2^ exhibiting a 16‐fold improvement compared to a pure CNT yarn‐based supercapacitor (2.3 mF cm^−2^). The fabricated device also exhibited excellent flexibility, as it maintained its capacitance at 100% even after being subjected to 150 cycles of bending.

CNT yarn is one of the most commonly used backbone materials among the yarn based supercapacitors. Strengthening and improving their conductivity is a crucial aspect toward developing reliable and strong wearable electronics. CNTs can be engineered and strengthened by electron and ion beam irradiation.^158^ Gamma irradiation of CNT yarns has been performed to improve the tensile strength and modulus of CNTs.^159^ Su and Miao developed a two‐ply yarn supercapacitor with gamma‐irradiated CNT (IR‐CNT) yarn infiltrated with PEDOT/PSS.^152^ Gamma irradiation improves the mechanical^159^ and electrical properties^160^ as well as the capacitance of the CNT yarn supercapacitor. The dimensions of the final strand of a single yarn of IR‐CNT@PEDOT/PSS along with PVA gel electrolyte was ≈35 µm. The morphology of the CNT yarn was restored even after the gamma irradiation and the expected outcome of an increase in mechanical strength and electrical conductivity was achieved. The IR‐CNT yarn exhibited typical EDLC behavior and provides 1.5 to 2 times enhanced gravimetric capacitance when compared to pure CNT yarn. A capacitance of 18.5 F g^−1^ at a current density of 0.1 A g^−1^ was achieved from the most improved IR‐CNT@PEDOT:PSS. The low capacitance of this two‐ply yarn supercapacitor compared to bulk nanocomposites is attributed to a lower mass fraction in the yarn. The main advantages of such a structure are the flexibility and essential mechanical strength to be woven into fabric. Cyclic folding tests proved that 98.8% of original capacitance could be retained even after 200 cycles of bending. Su and Miao developed an asymmetric two‐ply yarn supercapacitor with a CNT/metal oxide composite yarn as positive electrode and CNT yarn as negative electrode.^149^ Manganese dioxide (MnO_2_) which is known to have a high theoretical capacitance was chosen as the electrode material in this work. The asymmetric configuration of this structure helped to achieve an operating potential of 2 V. An energy density of 42 W h Kg^−1^ at a power density of 483.7 W Kg^−1^ was achieved. The energy density attained in this report is substantially higher than the typical supercapacitors. This is due to the high voltage accomplished for asymmetric geometry. The device was additionally tested for performance stability against cyclic charge–discharge under normal (retained 98% capacitance after 500 cycles) and bending conditions (retained 99.5% capacitance after 200 cycles). This demonstrates that this two‐ply yarn supercapacitor can be knitted or woven into fabrics without significant deterioration of its electrochemical performance.

A conventional supercapacitor houses metal foils as current collectors. These metal current collector electrodes enable efficient transport of charges produced by the active materials along the length of the supercapacitor. Even in the case of a linear supercapacitor, platinum filament core/CNT sheath yarn supercapacitor has been developed to improve the electrochemical performance (10.1 F g^−1^ for CNT to 86.2 F g^−1^ for Pt/CNT/PANI) and to scale up the length of the supercapacitor.^141^ Zhang et al. devised a new architecture of a multi‐ply yarn supercapacitor developed by investigating six different metal/alloy filaments (Pt, Au, Ag, AuAg, PtCu, and Cu) as current collectors for the CNT yarn.^156^ Metals/alloys like Cu and Pt–Cu were found to be the best candidates among all the different metal filament current collectors that were evaluated. The metal filament and the CNT yarn were twisted together to form an M + CNT yarn at a twist density of 150 T m^−1^, where M denotes one of the six metal filaments. A typical multi‐ply yarn supercapacitor was fabricated by twisting the M + CNT yarn with a PVA coating, one on top of the other. The metal filament integration to the CNT yarn significantly improves the current density. Maximum capacitances of ≈148 and ≈156 F g^−1^ were achieved with the PtCu and Cu filament integrated CNT yarns. The reason for the higher capacitance was attributed to the oxidation of Cu filament to copper oxide (CuO) at regions that are in contact with the PVA–H_3_PO_4_ gel electrolyte, and PtCu resulting in two active materials, CuO and CNT. The remaining unoxidized core of the metal functions was the current collector. This symmetric multi‐ply yarn supercapacitor can provide a voltage output up to 1.4 V that represents an increase of ≈20%. Even after 1000 cycles of folding and unfolding, the Cu + CNT supercapacitor had 97.5% capacitance retention making it reliable for wearable electronics.

#### 5.2.2 Fiber/Cable Type Supercapacitors

Even though yarn supercapacitors are made of CNTs as the active material, their performance is often dictated by the electrical conductivity of CNTs if they are used as both charge transporter and active material for energy storage. Therefore, an efficient metal current collector is introduced in a few recent architectures, as discussed in the previous section.^161^, ^162^, ^163^ Replacing CNT with metal oxide can lead to the development of pseudocapacitors with higher energy density.^164^ A major challenge for this approach is to make nanostructures of these psuedocapacitive materials in the form of fibers/cables. Once developed, these fibers can be stitched to make a fabric or can be woven into a textile, since they have good flexibility and mechanical stability. Like LIB fibers, fiber supercapacitors can have helically coaxial and parallel or twisted designs. Once structured into fibers or filaments, they can be weaved along with electrical connection filaments to charge and discharge the fibers. One of the early reports on the development of fiber type supercapacitors is reported by Wang and co‐workers.^165^ They fabricated a fiber electrode by growing ZnO nanowires on a Kevlar fiber. A similar fiber electrode was also developed with ZnO nanowires grown on PMMA fiber. Gold was deposited on ZnO nanowires to enhance the electrical conductivity. The fiber supercapacitor was assembled by entangling the PMMA fiber around the Kevlar fiber with the electrolyte in between.^143^ To improve the specific capacitance, the ZnO nanowires that were grown on the PMMA wires were electrochemically deposited with MnO_2_. The fiber supercapacitor provided a specific capacitance of 2.4 mF cm^−2^ in PVA/H_3_PO_4_ gel electrolyte. Since the fiber developed was flexible and a semi‐solid electrolyte was used, this configuration can be suitable for wearable energy storage devices.

**Figure 15:**
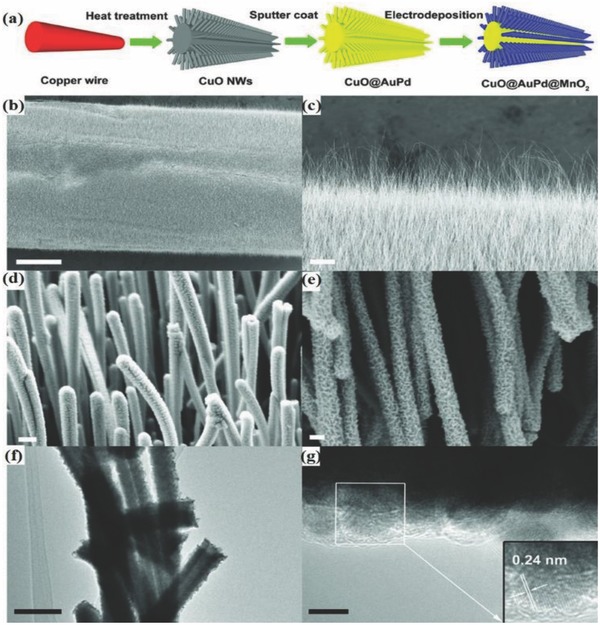
a) Schematic illustration showing the fabrication process of CuO@AuPd@MnO_2_ NWs. SEM image showing b) CuO NWs completely covering the copper wire. c) Vertically grown CuO NWs. d) AuPd nanoparticles that were conformally sputter‐coated onto each NW. e) Uniformly electrodeposited MnO_2_ onto NWs. f) TEM image of CuO@AuPd@MnO_2_ NWs. g) HRTEM image of CuO@AuPd@MnO_2_ NW. The inset is the enlarged HRTEM image of the rectangular area. Reproduced with permission.^166^ Copyright 2014, Wiley‐VCH.

Recently, a cable supercapacitor was developed by integrating energy storage and electrical conduction into a single cable by Thomas.^166^ A coaxial cable design named coax was fabricated with an inner electrical cable for electrical transmission and CuO nanowhiskers (CuONW) on the outside of the same cable for energy storage. CuONWs were developed by simply heating the Cu wire to 500 °C in the air. A copper foil constructed in the form of a tube with CuONW grown on the inside served as the second electrode. The two electrodes were glued together using PVA electrolyte with a thin porous polymer film in between. The region which stores charges consists of a high aspect ratio CuO@AuPd@MnO_2_ (AuPd–gold–palladium) core–shell NWs (**Figure** **15**). This novel design of energy storage on the outside of an electrical energy transmitting wire helps in saving space for applications, such as wearables, portable electronics, electric vehicles, etc. CuO prepared on the copper wire provides the advantage of reducing the leakage current because of its very low electrical conductivity. The active material MnO_2_ enables in achieving specific capacitance as high as 1376 F g^−1^. A solid‐state cable assembled exhibited an excellent mechanical stability with similar cyclic voltammetry (CV) curves upon bending from 0° to 180°. It retained 93.4% of the initial capacitance even after 100 bending cycles at about 180°. In addition, good cyclic performance was achieved retaining about 99% of its initial capacitance even after 5000 cycles at 100 mV s^−1^. These characteristics make the coax cables an ideal candidate for wearable electronics. In addition to its attractive energy storage capacity, the device can also be used for energy transmitting applications. It has also been demonstrated using CV curves and current–voltage (*I*–*V*) plots that energy transmission is not affected by energy storage and vice versa. The cables can be made in the form of very thin fibers and can be weaved into a matrix to use as a wearable energy transmitting as well as storage device.^167^

Another prudent approach to make cable supercapacitors with high energy density is to follow asymmetric configuration.^168^, ^169^, ^170^ In this design, two different active materials are used to achieve a larger voltage output. Senthilkumar et al. fabricated a cable‐type supercapacitor with β‐Ni(OH)_2_ as the cathode and AC as the anode.^171^ Copper and stainless‐steel wire‐like threads were used as current collectors. The active materials were fabricated into slurry and brushed on the current collectors. PVA–potassium hydroxide (KOH) gel electrolyte was dip coated on the active materials and inserted into a rubber tube with a gel electrolyte injected in between. A working voltage of 1.4 V was achieved with maximum energy density of 10.7 µW h cm^−1^ (9.8 W h Kg^−1^) at a power density of 169 µWh cm^−1^ (154 W Kg^−1^). The higher energy density achieved because of asymmetric configuration would be attractive to power those wearable devices that require higher power. Nagaraju et al. followed an eco‐friendly approach utilizing Cu fibers from electronic waste (e‐waste) as substrate to develop wearable fiber‐based hybrid supercapacitor device.^172^ An asymmetric fiber supercapacitor is assembled using two electrodes; NiO nanosheets (NSs)@CNTs@CuO nanowire arrays (NWAs) on Cu fiber electrode and an AC@carbon fiber (CF) electrode. The fabricated device exhibited a high specific capacitance of 93.42 F g^−1^ at a discharge current of 0.7 mA with high energy and power densities of 26.32 and 1218.33 W kg^−1^, respectively. The fiber device could also retain 83.6% of its initial capacitance after 2000 cycles in normal conditions and 94.5% when bended for 200 cycles. The fiber device connected in series was woven into a shirt to demonstrate its operation of powering an LED and an electronic display.

**Figure 16:**
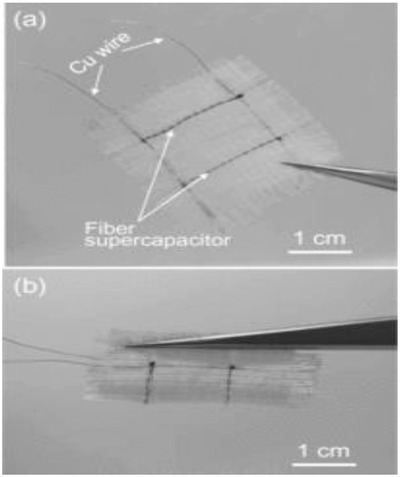
a,b) Photos of the textile embedded with two GF@3D‐G fiber supercapacitors in flat and bending states, respectively. Reproduced with permission.^147^ Copyright 2013, Wiley‐VCH.

Several successful cables and fibers for wearable applications were developed using carbon, CNT, and graphene as active materials for energy storage.^129^, ^133^, ^146^, ^173^, ^174^, ^175^ A polypyrrole (PPy)–MnO_2_–CNT–cotton thread‐based cable was developed using a three‐step process.^128^ In this approach, the cotton threads were initially made conductive by dip coating in single‐walled carbon nanotube (SWNT) ink followed by electrochemical growth of MnO_2_ nanostructures. The PPy film was deposited electrochemically on top of SWCNT coated cotton threads. A cable supercapacitor was assembled using two SWCNT–PPy–MnO_2_ cotton threads, one wrapped with uncoated cotton thread (separator), all inserted into a silicone pipe. A 0.5 m Na_2_SO_4_ electrolyte was added into the silicone pipe as the electrolyte. An advantage of this design is that the PPy film deposited on top of the MnO_2_ can work both as an electron collector and as a charge storage medium. These threads yielded a high areal energy density of 33 µW h cm^−2^ and a power density of 0.67 mW cm^−2^ with 96% capacitance retention after 3000 cycles. Further, it was demonstrated that a 2 m long fiber could be easily wound on a Teflon rod. Meng et al. developed a core–sheath fiber where a core of highly flexible graphene fiber is covered with a sheath of 3D graphene framework.^147^ It showed an energy density of 0.4–1.7 × 10^−7^ W h cm^−2^ and a power density of 6–100 × 10^−6^ W cm^−2^. The assembled fiber supercapacitor showed a stable capacitance of about 40 µF in the straight or a stretchable form for 500 cycles of bending. As shown in **Figure** **16**, a piece of textile is weaved using this core–sheath fiber. These fibers exhibited high flexibility and bending resistance that is highly necessary for wearable applications.

As in the case of Li‐ion batteries, a coil‐type fiber can sustain more mechanical strain than a typical fiber. A fiber asymmetric supercapacitor which can store and transport energy with a coil‐type architecture has been proposed and demonstrated by Yu et al.^176^ A copper wire with CuONWs grown on the outer side of the wire was electrochemically deposited with MnO_2_ (cathode). Carbon fiber with iron oxide@carbon (Fe_2_O_3_@C) core–shell structured nanorods grown by hydrothermal and heat treatment process was used as the anode. The coil‐type asymmetric fiber supercapacitor was assembled by wrapping the carbon fiber around the copper wire with a thin ion porous separator and LiCl/PVA gel electrolyte in between the structures. This coil‐type fiber supercapacitor exhibited high energy density (0.85 mW h cm^−3^) and excellent bending cycle stability (≈93.0% after 4000 cycles) and high rate capability (95.4%). This type of fiber is very useful in wearable applications where energy storage and energy transmission are required simultaneously.

#### 5.2.3 Screen Printed Supercapacitors

The fiber or cable supercapacitors discussed so far are necessary to be weaved or incorporated into fabric to be used as a wearable energy storage device. In addition, screen printed supercapacitors are proposed as an energy source, since they are lightweight, nontoxic, and nonflammable. Screen printing is a coating technique where ink to be coated is pushed using a squeegee on to the substrate, usually a fabric, through a screen mask with a desired pattern. Utilizing this technique wearable supercapacitors or batteries with high power and energy density can be achieved.^177^, ^178^, ^179^, ^180^ Techniques other than screen printing like ink‐jet printing^181^ and dip‐coating^182^ are also used for large‐scale electrode fabrication. In a fabric supercapacitor for wearable applications, mass loading of the active material per unit area plays an important role in maximizing the charges stored. The energy stored in textile supercapacitors must be measured in terms of the areal capacitance (F cm^−2^ since the average human body has a surface area of ≈1.5 m^2^. High gravimetric properties and areal capacitance go hand‐in‐hand in any energy storage device only when high mass loading of the active material is achieved.^183^, ^184^

Recently, screen printing using porous AC derived from coconut shells (YP17) was conducted by Jost et al.^185^ Additionally, various basic and complex arrays of fabric like cotton lawn, polyester microfiber, cotton twill, double knit with silver, and nylon neoprene were also studied. Carbon slurry for screen printing was prepared by mixing AC with 5 wt% Liquitex. Symmetric two‐electrode device was fabricated using PTFE separator and liquid electrolyte enclosed in a polypropylene bag. The electrochemical studies on the printed fabric showed that the capacitance achieved from the cotton lawn (a hydrophilic natural staple fiber) and polyester microfiber (a high wicking fiber with 10 µm diameter) was better than other fabrics. The cotton lawn was found to have a lower mass (6.8 mg cm^−2^) and resistance (3 Ω cm^2^) and similar capacitance (85 F g^−1^) when compared to polyester. The cycle test proved that the cotton lawn has just 8% loss in capacitance from the original structure even after 10 000 cycles. Due to these reasons, cotton lawn was chosen to be the best candidate as the backbone fabric. A major disadvantage of this work is its use of a liquid electrolyte that limits its safe operational range.

**Figure 17:**
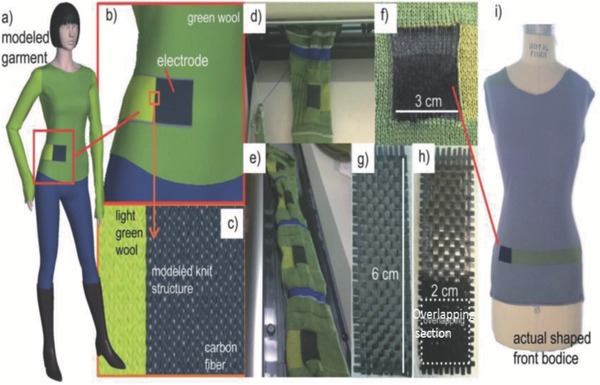
Seamlessly knitted and woven carbon fiber electrodes. a) 3D simulated model. b) Embedded textile supercapacitor in the shirt. c) Simulated knit structure rendered before fabrication. d) Carbon fiber current collector coming out of the knitting machine during fabrication. e) Four current collectors knitted at once. f) CF electrode screen printed with AC. Carbon fiber woven fabric g) before and h) after printing. i) A shaped front bodice knitted as one piece with a sample electrode made as a part of the textile. Reproduced with permission.^130^ Copyright 2013, RSC Publishing.

In a similar work, inactive cotton and polyester backbone from the previous work was replaced with highly conductive knitted and weaved CFs as the backbone of the supercapacitor.^130^ AC paint was used as the active material and silicotungstic acid solid polymer as the electrolyte. Intarsia knitting was followed for inserting the capacitive materials in the form of desired patterns within the textile while it is woven. CF electrodes with dimensions of 2 × 3 cm^2^ were woven into green wool with a layer of AC ink screen printed on them, as shown in **Figure** **17**. The machines used here for knitting were like 3D printers. Thus, they function as fast prototyping machines for mass scalability. Unlike the previous work, a solid electrolyte is used, which makes it feasible for wearable applications without any possible leaks. Knitted devices were found to have a high average mass loading of 12 mg cm^−2^ than the woven device with 6 mg cm^−2^ that reflected the higher areal capacitance achieved, 0.51 F cm^−2^ compared with 0.19 F cm^−2^ for the woven device. In general, electrodes with low mass loading provide high capacitance, as there is no restriction for electrolyte diffusion. However, in this case, the noncontinuous yarn structure in the woven device causes higher equivalent series resistance. Additionally, the long term mechanical stability of these devices needs to be tested before using it for wearable energy storage applications.

In a recent work, amorphous iron oxide hydroxide/MnO_2_ (FeOOH/MnO_2_) composite electrodes were screen printed on various substrates such as PET, paper, and textile to develop an all printed solid‐state flexible device.^186^ In this study, amorphous electrode material was preferred over the crystalline material owing to the fact that amorphous materials demonstrated moderate structural disorder facilitating faster ion diffusion by forming irregular surfaces providing high specific surface area and suitable pore size distribution.^187^, ^188^ The fabricated all‐solid state device exhibited a large specific capacitance of 350.2 F g^−1^ at 0.5 A g^−1^ current density while maintaining a good rate capability of 159.5 F g^−1^ even at high current density of 20 A g^−1^. It also exhibited an outstanding cycling stability, retaining 95.6% capacitance after 10 000 cycles.

Apart from these fibers, which show flexible and bendable nature for wearable applications, stretchability is an aspect to be considered as wearable energy storage devices undergo tensile stress during human body actions. To make a device stretchable, it is necessary to play with either material characteristic or structural characteristic. Micro‐supercapacitors (MSCs) provide shorter ionic diffusion and high utilization of their surface area due to structural design of parallel finger electrodes. These MSCs are usually thin in nature providing foldable and bendable characteristic without affecting their high performance. Such a device architecture is vital for developing on‐chip and wearable electronics.^189^ Upon introducing stretchable characteristic to this device architecture opens up a plenty of potential applications in wearable electronic devices. Chen and co‐workers developed highly stretchable MSCs based on suspended wavy structure of graphene microribbons.^190^ The wrinkled structure observed in these microribbons has the capability to release strain upon stretching by changing its shape with an out‐of‐plane bend, even with the active materials being stiff. The stretchable MSCs could retain up to 92% of its initial capacitance even after 5000 stretching cycles. In another interesting approach by the same group, editable and stretchable ultralong MnO_2_ nanowire (MNW) composite supercapacitors of desired shapes and stretchability were developed.^191^ Two obstacles to overcome developing editable supercapacitors are rigidity of electrodes and the instability of interfaces between different components of supercapctiors. These issues were addressed by developing a composite of MnO_2_ nanowires and CNTs sandwiched by nanocellulose fibers. The fracture strains of MNW are increased 10 times upon mixing with CNTs because of strong van der Waals forces among CNTs. The supercapacitor fabricated into a honeycomb‐like structure maintained capacitance retention of nearly 98% after 10 000 stretch and release cycles under reversible 400% tensile strain. Developing such a promising stretchable energy storage device shows its fascinating application in the future wearable electronics devices.

## 6 Self‐Reliant Energy Systems for Wearables

Wearable devices require the integration of various components that are flexible and durable in a single framework. As the technology advances, the wearable electronic equipment becomes smaller and smaller, and this demands the accommodation of multiple devices in a single framework. Any wearable device requires energy for operation, and a storage device is necessary to supply this energy. There have been tremendous advancements in the field of energy conversion and storage devices in the past few years.^192^, ^193^, ^194^, ^195^, ^196^, ^197^, ^198^, ^199^, ^200^, ^201^, ^202^, ^203^, ^204^, ^205^, ^206^, ^207^, ^208^, ^209^, ^210^, ^211^, ^212^, ^213^, ^214^, ^215^, ^216^, ^217^, ^218^ A combination of thin and flexible energy harvesting device and a portable storage device remains the best option for powering the wearable devices.

### 6.1 Integrated Triboelectric Nanogenerators‐Storage Devices

Triboelectricity is one of the key solutions for a sustainable and renewable alternative to any source of energy. It is not only environmentally friendly but also an efficient and low‐cost substitute for most of the renewable sources. Unlike the solar and wind energies, triboelectric energy can be harvested at any time of the day or night and is obtained by effectively converting the mechanical energy of the living or the working environment to electrical energy. The uncontrollable and fluctuating nature of the triboelectric output is one of the main disadvantages for its effective utilization. This issue can be addressed by storing the gathered energy in a device like battery or supercapacitor and getting the required power output on demand. To meet the ever‐growing demands of electronics in the modern world, enormous efforts are dedicated to developing integrated, portable, and wearable devices like electronic skin, portable displays, and on‐body sensors. These devices require a fully dependable source of energy that can be available for powering all the time. An integrated device consisting of a triboelectric nanogenerator and a thin film storage device is a good choice for satisfying the enormous demand for wearable and consumer electronics devices in the modern society.^219^, ^220^

**Figure 18:**
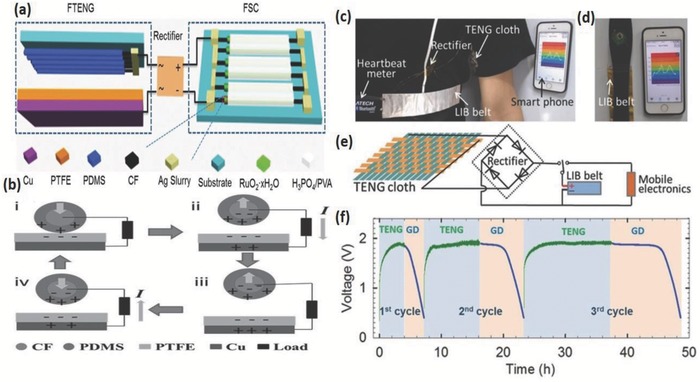
a) Schedule of integrating the fiber‐based supercapacitor triboelectric nanogenerator power system. b) Basic working principle of FTENG. Reproduced with permission.^221^ Copyright 2015, Wiley‐VCH. Self‐charging power unit. c) An optical image of TENG‐cloth that stores the energy with LIB belt, and then powers heartbeat meter strap, which has remote communication with a smart phone. d) The rear side of heartbeat meter, indicating that the LIB belt replaced its original coin cell. e) The equivalent electrical circuit of the self‐charging power system. f) The voltage profiles of the LIB belt charged by the TENG‐cloth and galvanostatically discharged (GD) at 1 µA for 3 cycles. Reproduced with permission.^222^ Copyright 2015, Wiley‐VCH.

Recently, Wang et al. were successful in fabricating a flexible and wearable energy harvesting and storage system combining a FTENG and flexible supercapacitor (**Figure** **18**).^221^ This is claimed to be the first prototype of a wearable device that can gather mechanical energy from human motion. The FTENG was fabricated by coating PDMS on carbon fiber electrodes as one of the triboelectric materials and PTFE as the second triboelectric layer with copper as the conducting film. When the PDMS and PTFE films come into contact, the electrons move from PDMS to PTFE surface. A negative charge is induced on the carbon fiber and positive charge on the copper film when the PDMS is detached from PTFE. As a result, the electrons flow from the copper film to the carbon fiber through a load until it reaches equilibrium resulting in a positive pulse as the output. As the polymers come close to each other, the induced charges on the copper film and the carbon fiber decrease. Now the copper film receives electrons from the carbon fiber and reaches a new equilibrium until the polymers are in contact with each other generating a negative current pulse. The FTENG when driven by a shaker motor under a periodic frequency of 2 Hz, a *V*_oc_ of 6.7 V was generated between the two electrodes that increases to 18 V at a frequency of 20 Hz. The change in *V*_oc_ was attributed to the variation in the stroke distance between the triboelectric electrodes due to the shaker. The *V*_oc_ and *I*_sc_ of FTENGs are far inferior compared to that of normal TENGs because of the smaller contact area, but many such connected fibers provide appreciable performance.

To accomplish better energy density, a textile‐TENG fabric integrated with a flexible LIB belt can be used.^222^ For the TENG, a conformal nickel (Ni) conductive coating on a polyester fabric that also serves as the flexible current collector for the LIB belt can be used. A parylene cloth‐wire that acts as the tribonegative part was woven into the tribopositive Ni coated polyester microwires. Electrons flow from the Ni‐coated wire to the parylene wire when they were brought in contact (contact‐electrification). This charge separation induces an electric potential difference between the two electrodes and facilitates the flow of electrons through the external circuit. *V*_oc_ increases when the contact is released and decreases when the contact is established by pressing the TENG. Two current pulses are thus generated in opposite directions corresponding to the contact and release of the electrodes. The equivalent electric circuit of the whole system is shown in Figure 18e. The so developed TENG‐LIB belt worn on the body was demonstrated as a self‐charging power source to operate a heartbeat monitoring system (Figure 18c,d). The stability of the power unit was confirmed further by charging and discharging battery with TENG‐cloth for 3 cycles (Figure 18f).

### 6.2 Photorechargeable Energy Storage Systems

The most promising of the renewable sources of energy is the solar energy that is abundant and readily available to harvest in most parts of the world. The incident light energy converted to electrical energy by a portable solar cell has to be stored in electrochemical storage devices like LIBs^198^, ^223^, ^224^, ^225^, ^226^, ^227^ or supercapacitors^18^, ^168^, ^228^, ^229^, ^230^, ^231^, ^232^ for effective usage.^233^ In many cases, lithium‐ion batteries^234^ are used where high energy densities and high operating voltages are required.^230^, ^235^, ^236^, ^237^ In applications requiring high power density, long cycle life, fast charge–discharge times, and a wide range of operating temperatures,^229^ a battery is often replaced with a flexible supercapacitor.^168^, ^230^, ^234^, ^237^, ^238^ In addition to the typical LIBs, there are many other rechargeable electrochemical storage devices such as lithium–sulfur batteries,^239^ lithium–oxygen batteries,^239^ and redox flow batteries.^240^

#### 6.2.1 Integrated Solar Cells and Li‐Ion Batteries

Wearable and flexible electronic devices can bring innumerable applications beyond imagination. Although considerable progress has been made in wearable solar cells, LIBs, and other similar power sources, there are still many issues like poor endurance of materials and mechanical deformations that hinder the performance of the integrated devices.

Efforts were carried out to integrate DSSCs with electrochemical storage devices over the past decade owing to their similar structures and nature of operations.^233^ Hence, a DSSC based configuration is adopted for most of the photocatalytic charging systems.^241^ The charges generated by the incident radiation are stored by a faradaic reaction (like LIB) or double layer charge storage (like supercapacitors) or a combination of both (hybrid supercapacitors). Recently, Guo et al. developed an integrated power pack with a series wound DSSC as the energy harvester and a LIB as the storage device on the same titanium (Ti) foil on which titanium dioxide (TiO_2_) nanotube (NT) arrays are grown on either side.^242^ The TiO_2_ NT arrays with increased surface area on the upper part are the electron collector for DSSC, while the lower part with TiO_2_ NT arrays is used for storing the harvested energy. The generated electrons from the dye molecules during irradiation will be injected into the conduction band of TiO_2_ NT arrays, while generated holes are being accumulated at Pt electrode. The electrons then react with lithium ions at the anode through the following chemical reaction, TiO_2_ + *x*Li^+^ + *x*e^−^ → Li*_x_*TiO_2_, charging the LIB part. Subsequently, free electrons released from cathode reaction, LiCoO_2_ → Li_1−_ *_x_*CoO_2_ + *x*Li^+^ + *x*e^−^, flow to the counter electrode of the DSSC to combine with holes on the Pt electrode. The solar cell provided a *V*_oc_ and *I*_sc_ of 3.39 V and 1.01 mA cm^−2^, respectively, by which the battery could be charged to 3 V in 8 min with a discharge capacity of 38.89 µA h and discharge density of 100 µA. The power pack exhibits a power conversion and storage efficiency of 0.82% and is claimed to be a potential power source for portable electronics.

**Figure 19:**
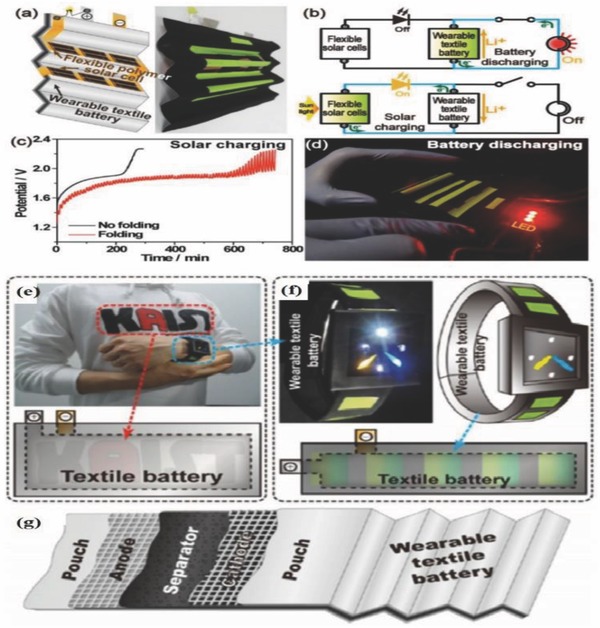
a) Schematic representation and photograph of the textile battery integrated with polymer solar cells. b) Equivalent circuits in the discharging and solar‐charging modes. c) Potential profiles of the textile battery in the presence and absence of the repeated folding–unfolding motions. d) A demonstration of battery operation. e) A photograph of wearable textile battery embedded in clothes together. f) Photograph of a watch with a wearable textile battery strap. g) A schematic illustration of the cell configuration of the wearable textile battery. Reproduced with permission.^119^ Copyright 2013, ACS Publications.

Other than DSSCs, energy conversion systems like organic/polymer solar cells, perovskite solar cells, and silicon‐solar cells can be used for the photocharging of storage devices. These kinds of devices can be considered as photovoltaic charging systems where the required charging voltage and current for the storage part is partially/fully provided by the solar cell part.^233^ Wearable textile batteries can be powered by polymer solar cells.^119^ The main components of the LIB, like current collector, separator, and binder, can be replaced with materials that can endure mechanical stress and strain. Out of these, the current collector is the most important material to be replaced, as it needs to sustain the entire mechanical deformations of the cell. Typically, to overcome this issue, current collectors are constructed out of conductive materials that are incorporated into textiles. For this, the conventional current collector can be replaced with nickel coated polyester yarn that can effectively withstand mechanical deformations. To have a better adhesion with the active materials and enhance the electrochemical, thermal, and mechanical properties, polyurethane based binder and separator can be used. The full‐cells fabricated in the wearable forms (like a watch strap or cloth (**Figure** **19**)) can exhibit similar electrochemical performances like that of the metal‐foil analogues even under severe mechanical deformations such as bending, folding, and unfolding actions. A flexible battery integrated with an array of polymer solar cells on the plastic substrates following this architecture provided a PCE of 5.49%, a voltage of about 2.4 V, and a stable current level of ≈5 mA.^119^

**Figure 20:**
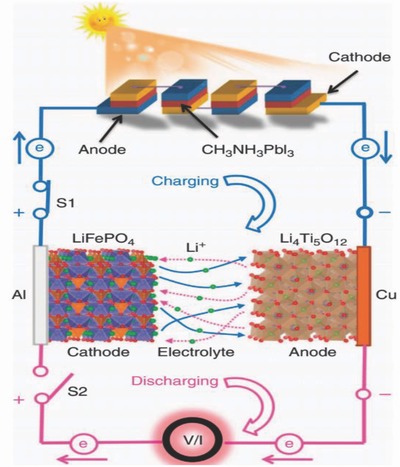
Schematic diagram of the fabricated system of PESC–LIB. Reproduced with permission.^226^ Copyright 2015, Springer Nature.

**Figure 21:**
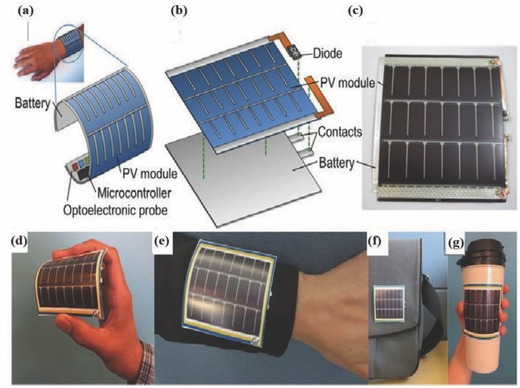
a) Illustration of activity‐tracking wristband concept containing flexible battery, PV energy harvesting module, and pulse oximeter components. b) Diagram and c) photograph of a flexible energy harvesting and storage system. d–g) Photographs of the device being flexed in the hand (d) and on various flexible and curved surfaces: jacket sleeve (e), bag (f), and travel mug (g). Reproduced with permission.^245^ Copyright 2016, Springer Nature.

Issues with efficient photocharging of the LIBs arise mainly because of the low current density and PCE of the thin film solar cells. This issue can be addressed by integrating multiple PESCs connected in series to directly charge LIBs.^226^ Photocharging of LIBs by PESCs connected in series is shown in **Figure** **20**. Flow of photogenerated holes and electrons into the cathode and anode of close circuited LIB leads to the charging of the battery. Discharge happens by turning S1 off and S2 on, involving backflow of lithium ions from anode to the cathode. The integrated device can possess very high cycling stability. It is an extremely challenging task to design a flexible battery without compromising the required charge/discharge capabilities and capacity retention for a wearable device. It is also important to note that the solar energy harvesters are a better choice for powering batteries of high capacities. They can deliver a power density in the range of 100 mW cm^−2^ in the outdoors and hundreds of µW cm^−2^ indoors when compared to the thermal and mechanical energy harvesters.^243^, ^244^ An optimized flexible power source for a wearable pulse oximeter (a health monitoring device) was recently designed and fabricated by Ostfeld et al. by integrating an amorphous silicon solar cell module with a LIB to power a pulse oximeter.^245^ As shown in **Figure** **21**a, pulse oximeter is fabricated as a single flexible device comprising the optoelectronic probe, microcontroller and flexible power source with photovoltaic (PV) module and battery. PV module and battery can be seen separately in Figure 21b,c. The flexibility of the entire device is evident from the Figure 21d–g. The LIB consists of flexible and thin current collectors made of stainless steel and nickel foils for the cathode and anode current collector electrodes, respectively, with lithium cobalt oxide on stainless steel and graphite on nickel as the active materials. The stainless steel and nickel current collectors were selected because of their high conductivity and capability of retaining their robust performance on repeated mechanical deforming actions. With a current density of 6.98 mW h cm^−2^, the battery retained a capacity of 90% at 3 C discharge rate, 99% even after 100 cycles of charge–discharge and 600 cycles of bending operations.

#### 6.2.2 Integrated Solar Cells and Supercapacitors

**Figure 22:**
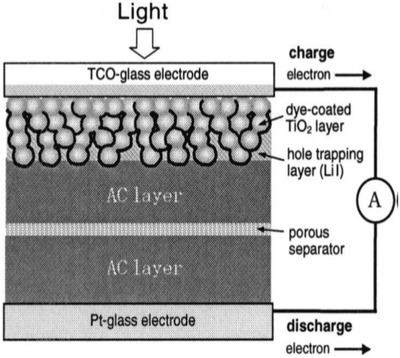
Schematic illustration for the two‐electrode, sandwich‐type multilayered photocapacitor. Reproduced with permission.^246^ Copyright 2004, AIP Publishing LLC.

The concept of a solar cell/supercapacitor integrated device was first proposed by Miyasaka and Murakami,^246^ which was named as a photocapacitor and was a three‐electrode dye‐sensitized photocapacitor (**Figure** **22**). Using this device, a capacitance of 0.69 F cm^−2^ was achieved on several charge–discharge cycles with a charging voltage of ≈0.45 V. A major disadvantage of this device was the switching of electrodes that requires a lot of energy and high fabrication cost.

**Figure 23:**
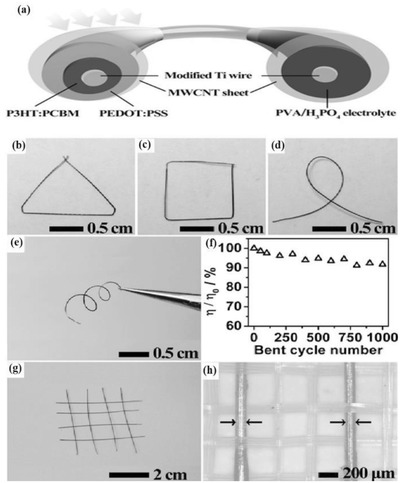
a) Schematic illustration to the structure of all‐solid‐state, coaxial, and integrated fiber device. b–e) “Energy fibers” being bent into various shapes. f) Photoelectric conversion and storage efficiency versus bending for 1000 cycles. η_0_ and η correspond to the entire efficiency before and after bending. g) “Energy fibers” being woven into a textile structure with each other. h) Photograph of two “energy fibers” being woven into a flexible aramid fibers textile. The arrow shows the “energy fiber.” Reproduced with permission.^247^ Copyright 2014, Wiley‐VCH.

The development of electrochemical capacitors in the thin film form has extended the realm of supercapacitors to flexible, portable, and wearable devices. An all‐solid‐state and flexible, polymer‐supercapacitor “energy fiber” developed by Zhang et al. was able to function as an integrated photovoltaic energy converter and energy storage device.^247^ The energy fiber consisted of a planar polymer solar cell in the radial direction and a supercapacitor in the coaxial architecture with a higher contact area (**Figure** **23**a). This configuration enables faster charge transport. The energy fiber has high flexible property and could be bent into various forms (Figure 23b–e). The total photoelectric energy conversion and storage was calculated by taking the product of the photoelectric conversion efficiency of the solar cell part and energy storage efficiency of the supercapacitor part. The energy conversion and storage efficiency of the energy fiber was more than 90% even after 1000 cycles of bending without proper sealing (Figure 23f). This efficient and stable performance is attributed to the coaxial architecture of the device. In addition, these “energy fibers” can be easily woven into fabrics, as shown in Figure 23g,h.

Recently, an all‐solid‐state device that integrates both solar energy harvesting and storage devices was demonstrated by Chai et al., which they called a tailorable fabric.^248^ In this device, an all‐solid‐state DSSC is combined with a fiber‐supercapacitor that provides ultrafast charging and sustains ultrahigh bending. The textile sample achieves a full charge in 17 s with the solar cell and discharges in 78 s at a discharge current density of 0.1 mA. All‐solid‐state DSSCs were interlaced with a fiber‐shaped photoanode and a counter electrode that forms a part of the titanium nitride (TiN) nanowire‐based symmetric fiber‐supercapacitor to make an energy sustainable smart fabric. In another approach of integrating DSSC and supercapacitor, ultrathin molybdenum disulphide (MoS_2_) nanofilms grown on TiO_2_ nanoparticles coated on CF were used as the electrodes in DSSC and supercapacitors.^249^ The self‐powering energy fiber showed a fast charging capability of 7s with an overall photochemical conversion efficiency of 1.8%.

**Figure 24:**
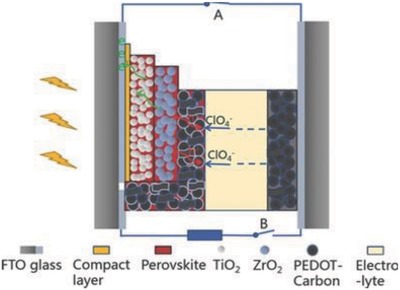
Schematic illustration and work mechanism of the photo‐supercapacitor device constructed based on a printable perovskite solar cell. Reproduced with permission.^251^ Copyright 2016, Wiley‐VCH.

Owing to the very high PCE offered by perovskite solar cells, a power pack combining a PESC and polypyrrole based supercapacitor was fabricated.^250^ An energy storage efficiency of 10% that is higher than the previous solar cell/supercapacitor combination was achieved. To improve the functionality, Xu et al. developed a printable PESC‐supercapacitor dual function device (**Figure** **24**).^251^ In this photo‐supercapacitor, a PEDOT–carbon electrode bridged the screen‐printed solar cell and the supercapacitor. The integrated device exhibited an overall efficiency of 4.70% and a remarkable energy storage efficiency of 73.77%. Although the device showed a lower efficiency due to the work function of the PEDOT–carbon electrodes, the research provided a scope for further improvement for developing wearable integrated energy devices.

A multifunctional power pack incorporating a PESC and a supercapacitor with common carbon electrodes was demonstrated by Liu et al.^252^ The device delivered an output voltage of 0.84 V from the supercapacitor part with an AM 1.5G illumination on a solar cell active area of 0.071 cm^2^. With this active area, the integrated device achieves an energy storage proportion (ratio of the energy stored in the supercapacitor to the total energy harvested by the solar cell) of 76% and a total energy conversion efficiency of about 5.26%. It was also observed that an instant overall output efficiency of 22.9% could be achieved when the supercapacitor is precharged to 1 V and a series connection is maintained between the supercapacitor and solar cell. This hybrid device is claimed to satisfy the needs for wearable and flexible electronics by effectively storing the harvested solar energy.

When an energy harvesting device is connected externally with an energy storage system using wires, the energy storage efficiency can be reduced due to the length of the wire connecting both the devices. Such losses can be avoided if the whole system is integrated into a single framework.^233^ In addition, this approach can save space and weight especially in applications like wearable fabrics for astronauts, soldiers, and first responders. Moreover, portability of energy devices can be substantially eased by developing these types of integrated energy packs. Currently, there are some efforts to integrate “back‐to‐back” devices with enhanced efficiency and flexibility for the wearable devices.^253^

**Figure 25:**
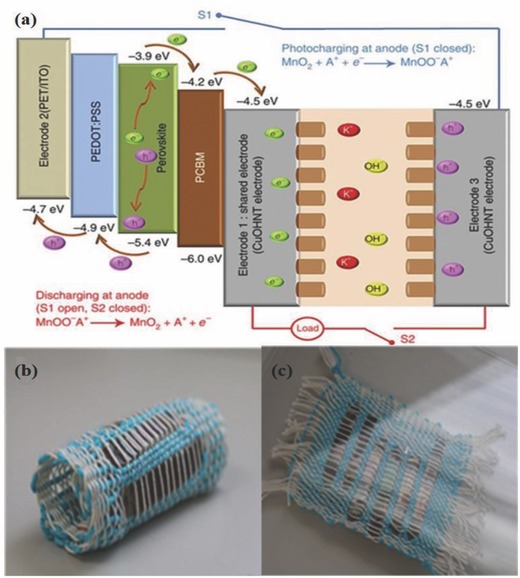
a) Charge transfer mechanism of the combination device. Reproduced with permission.^254^ Copyright 2016, Springer Nature. Photographs of b) the device woven into the fabric and c) device illuminated with sunlight.

Recently, our group has developed a flexible and low‐cost photovoltaic device integrated with supercapacitors on a copper ribbon that was woven into a fabric form to be used for charging wearable devices.^254^ Unlike the strategy of using two separate devices, a solar cell‐supercapacitor integrated device makes it easy to use as a wearable device capable of providing high energy density (1.15 mW h cm^−3^) and power densities (243 mW cm^−3^) per device. The ribbon developed works as a single bifunctional device, an energy harvester, and an energy storage device. A solvent‐assisted perovskite growth was adopted to fabricate the highly flexible sandwich PESC with PCE more than 10%. The copper ribbon was also used as a substrate for the growth of the copper hydroxide nanotubes (CuOH NT) for the supercapacitor. Two copper ribbons glued with the electrolyte, PVA containing KOH gel, worked as the supercapacitor. The fabricated PESC consists of PCBM as the ETL, perovskite as the photoactive layer, PEDOT:PSS as the HTL, and ITO on a PET sheet as the hole collector electrode. The working principle of energy harvesting and storing ribbons can be understood from **Figure** **25**. The shared electrode performs a dual function, aiding charge collection in the PSC and transferring it to the supercapacitor for storage. The positive electrode of the PSC is connected to the cathode of the supercapacitor through a switch. When the device is illuminated with sunlight, photocharging takes place, and the generated electrons in the perovskite layer are collected by the shared electrode and transferred directly to the anode of the supercapacitor. At the same time, the holes generated in the HOMO level of the perovskite move to the ITO through the hole‐transport layer. The charges reaching the anode and cathode of the supercapacitor are stored by a pseudocapacitive reaction. Finally, the connected load receives the power from the stored charge in the supercapacitor through the discharge process. The all‐solid‐state ribbon was highly flexible and self‐sufficient to be used as an excellent energy source for wearable applications.

## 7 Conclusions and Outlook

In this article, we tried to paint a picture of the current status of wearable energy devices that can be used to power the present and future wearable devices. A summary of the high‐performing wearable devices for harvesting, energy storage, and integrated devices is given in **Tables** **1**, **2**, **3**. It is to be noted that considerable progress has been accomplished even though serious research on the development of flexible and bendable fibers for wearable device applications has started only recently. Despite these great advances, there are still several roadblocks to overcome before deploying them for real‐world applications.

**Table 1:** Performance of wearable energy harvesting devices

Classification	Materials	*V*_OC_ [V]	*J*_SC_ [mA cm^−2^]	FF	Efficiency [%]	Ref.
DSSC	TCO free TiO_2_ film	0.707	5.07	0.676	2.4	41
	Oriented, crystalline ZnO nanowire array	0.61–0.71	5.3–5.85	0.36–0.38	1.2–1.5	42
	TiO_2_ nanowire array	–	–	–	5.38	44
	TiO_2_ nanotube array	0.670	15.46	0.648	6.72	46
	TiO_2_ nanocrystal film	–	–	–	7.19	47
	TiO_2_ micro cone array	0.702	16.036	0.717	8.07	48
	Hydrophilic and hydrophobic CNT	0.725	19.43	0.71	10.00	49
Fiber shaped polymer solar cell	Conducting polymer	0.607	11.9	53.8	3.87	60
	Polystyrene templated TiO_2_ nanocrystalline film	0.36	6.49	0.58	1.38	61
	P3HT:PCBM and PEDOT:PSS layers	–	–	–	1.08 and less than 3% variation after 200 bending cycles	62
Fiber shaped perovskite solar cell	Perovskite CH_3_NH_3_PbI_3_	0.664	10.2	0.487	3.3 and 95% PCE retained after 50 bending cycles	67
	Perovskite nanocrystals on CNT fiber	0.615	8.75	0.564	3.03 and 89% PCE retained after 1000 cycles	68
	Ti/c‐TiO_2_/meso‐TiO_2_/perovskite/spiro‐OMeTAD/Au	0.713	12.32	0.609	5.35	69
	Perovskite on CNT sheet	0.85	14.5	0.56	7.1	70
	Perovskite on TiO_2_	0.87	14.18	0.61	7.53	72

**Table 2:** Performance of wearable energy storage devices

Classification	Active material	Performance	Ref.
Lithium‐ion battery (LIB)	Ni–Sn and LiCoO_2_	Cell capacity: 1 mA h cm^−1^; Output voltage: 3.5 V	117
	CNT/silicon//CNT/LiMn_2_O_4_	Specific capacity: 106.5 mA h g^−1^; Voltage output: 3.4 V; Linear energy density: 0.75 mW h cm^−1^; Areal energy density: 4.5 mW h cm^−2^	118
	CNT/Li_4_Ti_5_O_12_//CNT/LiMn_2_O_4_	Energy density: 17.7 mW h cm^−3^; Power density: 0.56 W cm^−3^; Capacity retention: 97% after 1000 bending cycles, 84% after 200 stretching cycles	108
	Li_4_Ti_5_O_12_//LiFePO_4_	Specific capacity: ≈98 mA h g^−1^; Capacity retention: 91.8% after 40 cycles equivalent to 5500 deep folding–unfolding cycles	119
	Li_4_Ti_5_O_12_//LiCoO_2_	Maintains capacity density: ≈1.1 mA h cm^−2^; Output voltage: 2.5 V; reversible stretchability: 300%	51
	MWCNT/Li_4_Ti_5_O_12_//MWCNT/LiMn_2_O_4_	Specific capacity: 91.3 mA h g^−1^; capacity retention: 88% after 600% stretching	120
	CNT/Li_4_Ti_5_O_12_//CNT/LiMn_2_O_4_	Specific capacity: 92.4 mA h g^−1^; capacity retention: 92.1% after 100 cycles, 99% after 300 stretching cycles	121
	CNT/LiFePO_4_//CNT/Li_4_Ti_5_O_12_	Specific capacity: 110 mA h g^−1^	122
Yarn supercapacitor	CNT@PANI	Areal capacitance: 38 mF cm^−2^ at 0.01 mA cm^−2^; maintaining almost full capacitance after 150 bending cycles	157
	IR‐CNT@PEDOT/PSS	Specific capacitance: 18.5 F g^−1^ at 0.1 A g^−1^; Retained 98.8% of capacitance after 200 bending cycles	152
	CNT/MnO_2_	Specific capacitance: 12.5 F g^−1^ at 0.14 A g^−1^; retained 99.5% capacitance after 200 bending cycles. 42.0 W h kg^−1^ at a lower power density of 483.7 W kg^−1^, and 28.02 W h kg^−1^ at a higher power density of 19.250 kW kg^−1^	149
	Pt/CNT/PANI	Specific capacitance: 86.2 F g^−1^ at 5 mV s^−1^; energy density of 35.27 W h Kg^−1^ and power density of 10.69 kW Kg^−1^ at 100 mV s^−1^	136
	1. PtCu + CNT	1. Specific capacitance: 148 F g^−1^ at 10 mV s^−1^	156
	2. Cu + CNT	2. Specific capacitance: 156 F g^−1^ at 10 mV s^−1^; 97.5% capacitance retention after 1000 fold/unfold cycles	
Fiber/cable supercapacitor	Graphene fibers@3D‐G	Areal capacitance: 1.2‐1.7 mF cm^−2^; energy density: 0.4–1.7 × 10^−7^ W h cm^−2^; power density: 6–100 × 10^−6^ W cm^−2^	147
	MnO_2_ on ZnO nanowires	Areal capacitance: 2.4 mF cm^−2^ at 100 mV s^−1^; energy density of 2.7 × 10^−8^ Wh cm^−2^ and power density of 1.4 × 10^−5^ W cm^−2^	143
	Commercial pen ink	Areal capacitance: 11.9–19.5 mF cm^−2^; energy density: 1.76 × 10^−6^–2.7 × 10^−6^ W h cm^−2^ and a power density: 9.07 mW cm^−2^	144
	CuO@AuPd@MnO_2_ core–shell NWs	Specific capacitance: 1376 F g^−1^ at 5 mV s^−1^; 99% capacitance retention after 5000 cycles; 93.4% capacitance retention after 100 bending cycles. Power density: 0.55 mW h cm^−3^ and energy density: 413 mW cm^−3^	166
	β‐Ni(OH)_2_//AC	Specific capacitance: 481 F g^−1^ at 5 mV s^−1^; ASC device: 43.5 F g^−1^ at 5 mV s^−1^; 76% capacitance retention after 2000 cycles; energy density of 10.7 mW h cm^−1^ at a power density of 169 mW cm^−1^	171
	NiO NSs@CNTs@CuO NWAs/Cu//AC@CF	Specific capacitance: 93.42 F g^−1^; 83.6% capacitance retention after 2000 cycles; energy density of 26.32 W h Kg^−1^ at power density of 1218.33 W Kg^−1^	172
	PPy–MnO_2_–CNT	Areal capacitance: 1.49 F cm^−2^ at 1 mV s^−1^; 87% capacitance retention after 2000 cycles; High areal energy density of 33 µW h cm^−2^ at 0.67 mW cm^−2^ and a high areal power density of 13 mW cm^−2^ at 14.7 µW h cm^−2^	128
	Fe_2_O_3_@carbon//MnO2@CuO	Volumetric capacitance: 2.46 F cm^−3^ at 0.13 A cm^−3^; Good rate capability (95.4%); 98.5% capacitance retention after 200 bending cycles; Energy density of 0.85 mW h cm^−3^ at power density of 0.10 W cm^−3^	176
Screen printed supercapacitor	AC (YP17)	Specific capacitance: 85 F g^−1^ at ≈0.25 A g^−1^ in polyester microfiber and cotton lawn; 0.43 mF cm^−2^ at 5 mA cm^−2^ for both fabrics; 92% capacitance retention after 10 000 cycles	185
	AC (YP17)	Areal capacitance: 0.51 F cm^−2^ at mV s^−1^ and 88 F g^−1^ at 10 mV s ^−1^ for knitted CF; 80% capacitance retained after 200 cycles	130
	FeOOH/MnO_2_	Specific capacitance: 350.2 F g^−1^ at 0.5 A g^−1^; Good rate capability: 159.5 F g^−1^ at 20 A g^−1^; 95.6% capacitance retention after 10000 cycles	186

**Table 3:** Performance of wearable integrated devices

Classification	Material	Performance	Ref.
TENG–LIB	Polyester/Ni/paralene–Li_4_Ti_5_O_12_//LiFePO_4_	*V*_OC_: 50 V, *I*_SC_: 4 µA, Power density: 393.7 mW m^−2^. Discharge capacity: 81 mA h g^−1^, capacity retention: 85.4% after 30 times 180̊ folding. Integrated device: Powered a heartbeat meter for 3 cycles	222
TENG–SC	PDMS/PTFE–RuO_2_·*x*H_2_O@CF	*V*_OC_: 6.7 V at 2 Hz and 18 V at 20 Hz, peak current: 2 µA. Capacitance: 1.76 mF, capacitance retention: 96.4% after 100 bending cycles and 94% after 5000 cycles. Integrated device: charging current: 1.28 µA, voltage increased by 8 mV during 10 s	221
DSSC–LIB	TiO_2_ nanotubes–TiO_2_/LiCo_2_O_4_	*V*_oc_: 3.39 V, *I*_sc_: 1.01 mA cm^−2^, PCE: 0.82%. Charging: 3 V in 8 min Discharging capacity: 38.89 µA h	242
PSC–LIB	PCDTBT/PC_70_BM–Li_4_Ti_5_O_12_//LiFePO_4_	PCE: 5.49%, operating voltage: 2.4 V, supplying current ≈5 mA. Specific capacity ≈98 mA h g^−1^; Capacity retention: 91.8% after 40 cycles equivalent to 5500 deep folding–unfolding cycles	119
PESC–LIB	CH_3_NH_3_PbI_3_–Li_4_Ti_5_O_12_//LiFePO_4_	Specific capacity: 141.6 mA h g^−1^ PCE and storage efficiency: 7.80%	226
Silicon solar cell–LIB	Amorphous Si–graphite//LiCo_2_O_4_	Specific capacity: 1.8 mA h cm^−2^; Energy density: 383 Wh L^−1^; capacity retention ≈99% after 100 cycles, 600 bending cycles	245
DSSC–supercapacitor	TiO_2_ with Ru complex dye–AC	Charging voltage > 0.45, capacitance: 0.69 F cm^−2^	246
	ZnO NW–TiN NW	Tailored photoanode: *I*_sc_ ≈ 0.21 mA, *V*_oc_: 0.41 V. Specific capacitance: 2.28 mF cm^−2^ at 0.1 V s^−1^; rate capability: 37% at 10 V s^−1^; capacity retention: ≈87.5% after 5000 cycles, ≈98% after 2000 bending cycles. Charging: 1.2 V in 17 s	248
PSC–supercapacitor	P3HT:PCBM/PEDOT:PSS/MWCNT–MWCNT	PCE: 1.01%. Specific capacitance: 0.077 mF cm^−1^, energy density: 1.61 × 10^−7^ W h cm^−2^. η = 0.79%	247
PESC–supercapacitor	CH_3_NH_3_PbI_3_–polypyrrole/MWCNT	*V*_OC_ = 0.974 V, *J*_SC_ = 19.90 mA cm^−2^, FF: 0.7, PCE: 13.6%. Areal capacitance: 572 mF cm^−2^ at 1 mA cm^−2^, rate capability: 82% retention at 15 mA cm^−2^. High output voltage: 10 V, η = 10%	250
	CH_3_NH_3_PbI_3_–PEDOT/carbon	*V*_OC_: 0.71 V, *J*_SC_: 18.62 mA cm^−2^, FF – 0.48, PCE: 6.37%. Areal capacitance: 12 mF cm^−2^, capacitance retention: 95% after 2000 cycles. Charging: 0.70 V in 7 s, η_overall_: 4.7%, η_storage_: 73.77%	251
	CH_3_NH_3_PbI_3_–carbon/MnO_2_	*V*_OC_: 0.96 V, *J*_SC_: 15.7 mA cm^−2^, FF: 0.52, PCE: 7.79%. Areal capacitance: 61.01 mF cm^−2^, capacitance retention: 96.2% after 5000 cycles. η_overall_: 5.26%, η_storage_: 67.5%	252
	CH_3_NH_3_PbI_3_–MnO_2_	*V*_OC_: 0.96 V, *J*_SC_: 16.44 mA cm^−2^, FF: 0.66, PCE: 10.41%. Areal capacitance: 67.78 mF cm^−2^, capacitance retention ≈91% after 10 000 cycles and 98% after 100 bending cycles. Energy density: 1.15 mW h cm^−3^, power density: 243 mW cm^−3^	254

In view of practical applications, it is important to measure and optimize the mechanical properties of materials used for making these devices. Such measurements using the nanoindentation, microindentation, and macroindentation techniques ensure the mechanical robustness and stability of these materials for bending and stretching actions. The hardness, Young's modulus, strain‐rate sensitivity, and activation volume of the devices should be tested. The elastic and plastic properties of such materials can also be assessed by uniaxial‐tensile and compressive measurements.

For fiber solar cells, the best efficiency attained is not near to the efficiency achieved for other geometries like planar solar cells, although a straightforward comparison of both kinds of devices for the same active area is complicated. Additionally, fabricating fiber solar cells on a commercial scale is not an easy task especially, since the different layers need to be deposited cautiously. Dip coating is a feasible method to deposit the various layers on a large scale. To accomplish this goal, organic or hybrid materials such as perovskite are a better choice than Si. However, the lifetime of these materials in an open environment is still a challenge. However, there are a few reports in which PESCs are stable for 1000–2500 h of continuous simulated solar illumination.^255^, ^256^ New encapsulation methods using photocurable fluoropolymers^257^ and adopting perovskite materials modified with inorganic materials like cesium and rubidium^77^, ^78^, ^79^ are other feasible options to improve the overall stability and efficiency of these solar cells. This represents a clear opportunity, since the service time of the wearable devices is typically less than 5 years.

Nanogenerator fibers are considered as very promising devices for energy harvesting applications, especially making wearable fabrics. Since they can generate energy from body movements, they are a good alternative to the solar cells. Recently, this technology has considerably been evolved in terms of generated energy. However, presently, the energy derived is substantially low to be used for changing many of the advanced wearable devices applications. Because of the ease of device fabrication and the absence of any toxic materials, this is a very adoptable technology for wearable applications once the energy density is further improved.

Presently, LIB fibers offer high flexibility and weavability to make wearable fabrics. Although energy density achieved by LIBs is better than other devices such as supercapacitors, it needs to be improved further to be used as stand‐alone units (i.e., not attached to energy harvesting units). To further improve the energy density of the LIBs, a feasible approach is to use Si as the active material instead of carbon‐based materials. However, the charge–discharge cycle life may be compromised because of the mechanical strain caused by the expansion and contraction of Si. For LIBs, another important limitation is the use of an organic electrolyte in high‐performing devices that may be harmful to the human body in case of a leakage at any stage of its service. Therefore, an alternative is a supercapacitor that uses an aqueous electrolyte. For symmetric supercapacitor fibers, the power density is high, but an obvious limitation is the inferior energy density even though the charging is very fast. A natural substitute is an asymmetric supercapacitor or a hybrid battery–supercapacitor fiber.

It is our outlook that the energy devices to be developed for wearable energy fabrics should be capable of simultaneously harvesting and storing energy. Although there are a few interesting studies targeted to achieve this goal, they are currently limited by their inconsistencies in the electrical and electrochemical performances. The limitation of the inferior energy density of LIB or supercapacitor fibers can be rectified by integrating them with an energy harvesting device like a solar cell or a triboelectric nanogenerator. In the case of an integrated harvesting and storage device, the energy harvested can be stored in the storage device and can be continuously tapped by the wearable devices during its operations. The low energy density of the storage device is compensated by the energy harvesting capability of the integrated devices. A limitation though is that, if the solar cell is used as the harvesting device, the energy cannot be harvested during night time or when it is not exposed to sunlight.

A triboelectric generator can generate energy from the body movements, but the energy generated is much lower than that from solar cells. A combination of these two devices may be a better option. Another important aspect often overlooked is the shadowing effect of solar fibers when the fibers are weaved into a piece of textile. This can reduce the efficiency of the solar cell to a considerable extent. Therefore, a smart weaving approach should be developed to minimize shadowing effect when fibers are made into a fabric form. Very recently, Liu et al. used a mutual electrode to integrate a silicon solar cell with a TENG that can effectively harvest energy from the sunlight and raindrops.^258^ Thus, in the absence of sunlight during the rainy days, energy could be gathered from the raindrops. We believe that an integrated device is not going to completely replace the currently used batteries, but increase the time a device can be kept on charging or reduce the weight of the batteries carried by military personnel, astronauts, first responders, and other field workers.

For all the wearable devices, cleaning and safety of the fabric is an important aspect. These devices should be wet or dry‐cleaned. Therefore, the fibers developed should be amenable to the cleaning procedure followed for a given fabric. In addition, the safety of these fibers is a major concern especially when Li‐ion batteries are used. All the components used in wearable devices should be nonflammable. Moreover, it is important to consider their biocompatibility and mechanical reliability before proposing for a wearable energy device application. Finally, each component of the wearable device should be compatible with each other to perform in a synchronized manner without compromising the efficiency.

To summarize, substantial progress has been accomplished recently with respect to the fiber‐based energy devices that can be used to make wearable fabrics to charge electronic devices. In many reports, it has been shown that these fibers can be weaved into fabric form and can be used to power devices. We, along with other fellow researchers, are working toward the development of a fully functional fabric that can satisfy the requirements of a fabric that can be worn and be able to power any wearable devices connected to it in the near future.

## Conflict of Interest

The authors declare no conflict of interest.
